# Altered plastidial fatty‐acid condensation is associated with changes in membrane composition and postembryonic leaf development in Arabidopsis

**DOI:** 10.1111/tpj.71243

**Published:** 2026-11-06

**Authors:** Evan R. Angelos, Amirreza R. Bagh‐abrishami, María Fernanda Gómez‐Méndez, Katharina Gutbrod, Andrea A. Zanini, De Fan, Hee‐Seung Choi, Liping Zeng, Jiaxuan Cheng, Bailong Zhang, Meng Chen, Tessa M. Burch‐Smith, Peter Dörmann, Katayoon Dehesh

**Affiliations:** ^1^ Institute for Integrative Genome Biology and Department of Botany and Plant Sciences University of California Riverside CA 92521 USA; ^2^ Institute of Molecular Physiology and Biotechnology of Plants (IMBIO) University of Bonn 53115 Bonn Germany; ^3^ Donald Danforth Plant Science Center St. Louis MO 63132 USA; ^4^ State Key Laboratory of Aridland Crop Science Lanzhou Gansu China; ^5^ Ministry of Education Key Laboratory of Cell Activities and Stress Adaptations, School of Life Sciences Lanzhou University Lanzhou China; ^6^ Present address: Department of Life Sciences Pohang University of Science and Technology Pohang Republic of Korea

**Keywords:** plastidial fatty‐acid biosynthesis, KASII, membrane remodeling, chloroplast architecture, mesophyll organization, gibberellin, light‐responsive development, *Arabidopsis thaliana*

## Abstract

Membrane lipid composition is fundamental to organelle structure and environmental adaptation, yet how plastidial fatty acid metabolism influences developmental plasticity remains poorly understood. We examined the effects of sustained elevation of the plastidial fatty‐acid enzymes KASII (β‐ketoacyl‐ACP synthase II) and SSI2 (Δ9 stearoyl‐ACP desaturase 2) in *Arabidopsis thaliana* using lipidomic, ultrastructural, cellular, molecular, and physiological analyses. KASII overexpressing plants (KASIIox), but not SSI2 overexpressing plants (SSI2ox), increased carbon 18‐containing lipid species and exhibited reduced plastidial galactolipids together with increased extraplastidial phospholipids. KASIIox plants displayed enlarged chloroplasts with disrupted thylakoid organization, impaired palisade differentiation, altered mesophyll patterning, and reduced seed coat mucilage. Under low light, KASIIox plants failed to exhibit the characteristic petiole elongation observed in control and SSI2ox plants, accompanied by altered phyB and PIF4 protein abundance. Exogenous gibberellin intensified, whereas inhibition of gibberellin biosynthesis partially alleviated KASIIox leaf and cellular phenotypes. Together, these findings associate sustained KASII elevation with changes in membrane lipid composition, chloroplast architecture, light‐responsive growth, and postembryonic leaf development, supporting a link between plastidial fatty‐acid metabolism and developmental responses to light and gibberellin availability.

## Significance Statement

Membrane lipid composition is fundamental to organelle structure and environmental adaptation, yet how plastidial fatty‐acid metabolism influences developmental plasticity remains poorly understood. Here we find that sustained elevation of the plastidial fatty‐acid enzyme KASII disrupts chloroplast architecture, light‐responsive growth, and postembryonic leaf development, and supports a link between plastidial fatty‐acid metabolism and developmental responses to light and gibberellin availability.

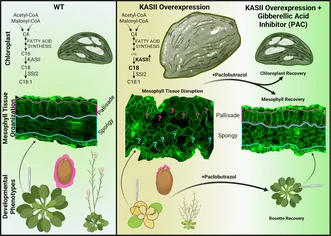

How organelle metabolism relates to whole‐plant development remains a fundamental question in plant biology. Our study shows that perturbations of plastid fatty‐acid metabolism are not developmentally equivalent: Sustained elevation of KASII, unlike SSI2, is associated with striking changes in postembryonic development and environmental responsiveness. This specificity identifies KASII‐associated fatty‐acid metabolism as a distinct metabolic state linked to developmental plasticity rather than a general consequence of altered fatty‐acid metabolism. These findings extend the biological significance of plastid fatty‐acid metabolism beyond lipid production and provide a foundation for understanding how specific organelle metabolic states are associated with plant development and environmental responses.

## INTRODUCTION

Plants continuously remodel their architecture to accommodate fluctuating environmental conditions. This developmental plasticity requires coordination between metabolic state and signaling networks that integrate organelle function with tissue specification and growth responses. Plastids occupy a central position in this integration. Beyond supplying carbohydrates, amino acids, isoprenoids, and fatty acids, plastids generate retrograde signals that modulate nuclear gene expression and developmental programs (Chan et al., 2016; de Souza et al., 2017; Jiang & Dehesh, 2021). Among these signals, the methylerythritol cyclodiphosphate (MEcPP) intermediate of the 2‐C‐methyl‐D‐erythritol 4‐phosphate (MEP) pathway has emerged as a key plastid‐derived metabolite that integrates stress perception with transcriptional and physiological responses (Xiao et al., 2013; Zeng et al., 2022; Zeng et al., 2024).

While MEcPP has been shown to coordinate stress signaling and growth modulation, the plastidial protein targets through which it interfaces with metabolic and structural processes remain incompletely defined. In the preliminary work presented in this study we probed potential plastidial effectors of MEcPP using limited proteolysis–mass spectrometry (LiP‐MS) (Pepelnjak et al., 2020), we detected signatures of conformational change consistent with the association of this metabolite with the fatty‐acid condensing enzyme β‐ketoacyl‐ACP synthase [KASII (FAB1)] and the Δ9 stearoyl‐ACP desaturase SSI2 (FAB2). This unexpected connection between a retrograde signaling intermediate and core fatty‐acid biosynthetic enzymes suggested that plastid acyl‐chain production itself might participate in stress‐responsive and developmental regulation. We therefore asked whether altering the abundance of these enzymes, thereby modifying plastid acyl‐chain output, would reshape membrane composition and influence organelle organization and environmentally regulated morphogenesis.

De novo fatty‐acid synthesis (FAS) occurs almost exclusively in plastids and generates the acyl chains that populate all cellular membranes (Li‐Beisson et al., 2013; Ohlrogge & Browse, 1995). Sequential condensation reactions yield 16:0‐ACP, which can undergo further elongation by KASII to produce 18:0‐ACP, followed by desaturation by SSI2 to generate 18:1‐ACP. In plants, these acyl chains are partitioned between two interconnected lipid assembly routes: the plastidial ‘prokaryotic’ pathway producing galactolipids and plastidial lipids such as monogalactosyldiacylglycerol (MGDG), digalactosyldiacylglycerol (DGDG), and phosphatidylglycerol (PG), which are major components of thylakoid membranes, and the endoplasmic (ER)‐based ‘eukaryotic’ pathway producing phospholipids including phosphatidylcholine (PC), phosphatidylethanolamine (PE), and phosphatidylinositol (PI) (Benning, 2008; Hölzl & Dörmann, 2019). Lipid transport from ER to plastids mediated by the trigalactosyldiacylglycerol (TGD) complex further integrates plastid and ER membrane pools, such that the lipid backbones employed for galactolipid synthesis are derived from both the prokaryotic and eukaryotic pathways (Awai et al., 2006; Benning et al., 2007; Fan et al., 2015; Xu et al., 2008). Although the enzymology and trafficking routes are well characterized, whether quantitative shifts in plastid acyl‐chain output can redefine membrane identity, and thereby influence organelle architecture and developmental signaling, remains unresolved.

Membrane composition is not merely structural; it determines bilayer thickness, curvature stress, and protein–lipid interactions that shape organelle architecture and signaling capacity. Thylakoid organization depends critically on galactolipid balance, and perturbations in lipid composition destabilize grana stacking and photosynthetic membrane structure (Dormann & Benning, 2002; Kobayashi, 2016; Yu et al., 2020). More broadly, membrane properties influence the functions of membrane‐associated processes including receptor localization, vesicle trafficking, and hormone transport (Grebe et al., 2003; Munnik & Testerink, 2009; Sallese et al., 2006; Zhao et al., 2010). Consequently, hormone‐regulated developmental outputs are particularly sensitive to membrane organization as they often rely on receptor dynamics and polarized transport pathways to actuate their signaling functions.

Gibberellins (GAs), together with auxin and light signaling pathways, play central roles in coordinating cell expansion, tissue differentiation, seed coat development, and adaptive growth responses (Cao et al., 2006; Daviere & Achard, 2013; Gallego‐Bartolomé et al., 2012; Sun & Gubler, 2004; Ubeda‐Tomas et al., 2009). GA signaling also interfaces extensively with developmental and environmental response networks to shape organ growth, tissue patterning, and developmental plasticity (Cheminant et al., 2011; de Lucas et al., 2008; Feng et al., 2008; Schwechheimer, 2011).

Light‐regulated morphogenesis provides a sensitive developmental context in which such membrane‐dependent processes may operate. Phytochrome B regulates phytochrome‐interacting factors (PIFs) to control auxin biosynthesis and elongation growth under reduced irradiance (Franklin & Quail, 2009; Hornitschek et al., 2009; Hornitschek et al., 2012; Leivar & Monte, 2014; Leivar & Quail, 2011), linking light perception to developmental outputs (Casal, 2012; Cheminant et al., 2011; de Lucas et al., 2008; Feng et al., 2008; Schwechheimer, 2011). Yet how membrane lipid state intersects with these integrated hormone and light signaling modules to determine developmental architecture and environmentally responsive growth remains unknown.

Here, we test whether plastidial fatty‐acid chain length output links membrane composition to developmental plasticity. KASII overexpression redirects lipid metabolism toward C18 acyl species, reconfiguring plastidial and ER membrane composition and consequently altering chloroplast organization, mesophyll architecture, seed coat mucilage deposition, adaptive low‐light growth, and GA‐associated responses. By contrast, SSI2 overexpression has comparatively minor effects. These findings support a link between plastidial fatty‐acid chain length, tissue patterning, hormone‐responsive development, and environmental adaptation.

## RESULTS

### Sustained elevation of plastidial fatty‐acid condensation and desaturation differentially affect plant growth and chloroplast ultrastructure

To identify plastidial targets of the retrograde metabolite MEcPP, we performed limited proteolysis–mass spectrometry (LiP‐MS), which detects ligand‐induced conformational changes. This analysis revealed discrete MEcPP‐responsive regions in the fatty‐acid enzymes KASII and SSI2 (Figure S1A,B): two in SSI2 (aa 99–119, 301–312) and one in KASII (aa 356–376). Docking analysis guided by the LiP‐MS derived peptides supported the plausibility of these potential interaction sites in solvent‐accessible domains near the catalytic cores (Figure S1).

Because recombinant KASII could not be recovered in a soluble form suitable for biochemical validation, and KASII loss‐of‐function mutants are lethal (Pidkowich et al., 2007), we instead examined the in vivo effects of elevated KASII or SSI2 abundance. To this end, β‐estradiol (β‐EST)‐inducible KASII (inKASII) and SSI2 (inSSI2) lines were generated in the background of the previously established dexamethasone (DEX)‐inducible HDS RNAi system (HDSi), in which DEX treatment suppresses HDS expression and promotes MEcPP accumulation (Jiang et al., 2018). Under all induction regimes, seedling morphology remained largely comparable to controls, apart from chlorosis in newly emerging leaves following DEX treatment, consistent with the previously reported phenotype of elevated MEcPP levels resulting from HDS silencing (Figure S2) (Jiang et al., 2018; Wang et al., 2022; Zeng et al., 2024).

MEcPP quantification confirmed similar metabolite accumulation across all genotypes following DEX induction, and β‐EST‐mediated induction of KASII or SSI2 did not further alter MEcPP abundance (Figure S2; statistical summary in Table S1). Immunoblot analyses further verified robust induction of KASII–HIS and SSI2–FLAG proteins, while endogenous HDS abundance was comparably reduced in all DEX‐treated backgrounds (Figure S2), confirming equivalent silencing efficiency.

Because MEcPP accumulation activates ER unfolded protein response (UPR) signaling (Walley et al., 2015; Wang et al., 2017), we then tested the effects of β‐EST‐mediated induction of KASII or SSI2 on MEcPP‐dependent transcriptional reprogramming by monitoring expression of the ER stress markers *BiP3* and *sbZIP60*. As expected, DEX treatment induced both markers; however, β‐EST treatment attenuated this response across all genotypes, including the HDSi control line (Figure S2), indicating that the suppression is independent of KASII or SSI2 induction. Prolonged β‐EST exposure (i.e., germination on β‐EST‐containing medium rather than post‐germination induction) caused generalized growth retardation, including in HDSi controls, indicating effects beyond transgene induction (Figure S2). However, growth defects were more severe in KASII‐expressing seedlings and, to a lesser extent, SSI2‐expressing seedlings, suggesting additional enzyme‐specific developmental effects. To avoid the pleiotropic effects of prolonged β‐EST treatment and to directly assess the consequences of elevated enzyme abundance, we generated constitutive KASII and SSI2 overexpression lines. Elevated transcript abundance was confirmed in independent KASIIox and SSI2ox lines (Figure S3). Both KASIIox and SSI2ox seedlings were smaller than HDSi controls (hereafter referred to as the control line), but KASIIox plants exhibited a markedly stronger phenotype, including smaller true leaves, and pronounced chlorosis (Figure 1A; Figure S3B). The developmental effects were tissue specific, KASII overexpression had little effect on primary root elongation whereas SSI2ox lines showed modest reductions in root growth (Figure 1B; Figure S3). Thus, despite their sequential roles in plastidial fatty‐acid biosynthesis, elevated KASII and SSI2 abundance produced distinct developmental outcomes.

**Figure 1:**
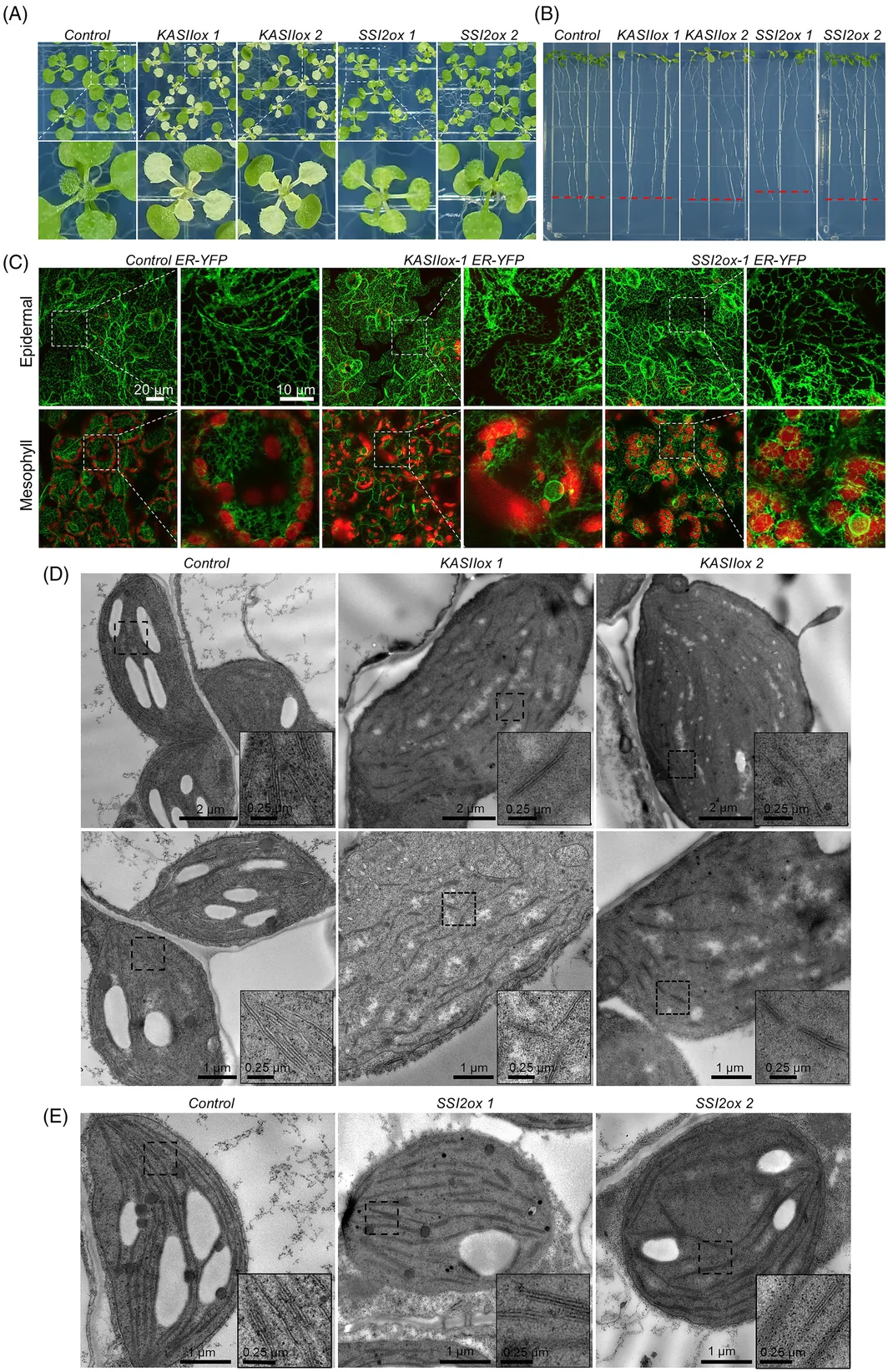
Plastidial fatty‐acid condensation remodels plant morphology, organelle organization, and chloroplast ultrastructure. (A) Representative images of 14‐day‐old *Arabidopsis thaliana* seedlings (top) and magnified views of representative seedlings (dashed boxes; bottom) from control, KASII overexpression (KASIIox 1, KASIIox 2), and SSI2 overexpression (SSI2ox 1, SSI2ox 2) lines. KASII overexpression markedly alters plant architecture, resulting in chlorotic, developmentally distorted leaves with protruding hydathodes and irregular wrinkled lamina, accompanied by modest reductions in overall seedling size, whereas SSI2 overexpression produces comparatively mild morphological effects. Statistical analysis of leaf size is provided in Figure S3. (B) Representative root phenotypes of the indicated genotypes grown under standard conditions. Red dashed lines indicate average primary root length. Red line indicates average primary root length; quantification and statistical analysis provided in Figure S3. (C) Confocal imaging of endoplasmic reticulum organization (ER–YFP, green) and chloroplasts (chlorophyll autofluorescence, red) in epidermal (top) and mesophyll (bottom) cells of third true leaves. Dashed boxes indicate regions shown at higher magnification. Scale bars: 20 μm; enlarged panels, 10 μm. A total of 10 leaves were sampled from two independent experiments; representative max intensity projections are shown. (D) Transmission electron microscopy (TEM) analysis of chloroplast ultrastructure in control and KASII overexpression lines. KASII overexpression results in enlarged chloroplasts with disrupted thylakoid organization, including reduced grana stacking and increased stromal thylakoid prevalence. Two representative chloroplasts are shown per genotype, with the lower row shown at increased magnification. Dashed boxes indicate regions magnified in the inset panels. Scale bars: main panels, 1–2 μm; insets, 0.25 μm. (E) TEM analysis of chloroplast ultrastructure in mesophyll cells from control and SSI2 overexpression lines (SSI2ox 1, SSI2ox 2). In contrast to KASII overexpression, chloroplast morphology, grana stacking, and thylakoid organization are largely unaffected by SSI2 overexpression. Dashed boxes indicate regions magnified in the inset panels. Scale bars: main panels, 1–2 μm; insets, 0.25 μm.

Phenotypic analysis throughout development revealed markedly stronger effects of KASII overexpression than SSI2 overexpression. While SSI2ox plants exhibited only modest reductions in leaf growth and mild leaf curling, KASIIox plants displayed severe dwarfing, compact rosettes, delayed greening, enhanced leaf curvature, and additional morphological abnormalities affecting reproductive organs (Figure S4A–D). To determine whether these developmental phenotypes were associated with altered subcellular organization, ER–YFP reporters were introduced into control, KASIIox, and SSI2ox backgrounds. Confocal imaging revealed largely comparable ER morphology across genotypes; however, plastid organization was markedly altered in KASIIox plants, which contained enlarged and irregular chloroplasts, whereas plastids in SSI2ox lines resembled those of controls (Figure 1C).

Transmission electron microscopy further demonstrated distinct ultrastructural consequences of sustained plastidial fatty‐acid enzyme elevation. Chloroplasts in control and SSI2ox mesophyll cells retained canonical organization, with densely stacked grana and well‐defined stromal lamellae, whereas KASIIox chloroplasts exhibited extensive architectural remodeling, including reduced grana stacking and expansion of stromal thylakoid membranes (Figure 1D,E).

Collectively, these findings demonstrate that sustained elevation of plastidial fatty‐acid condensation capacity profoundly reprograms chloroplast organization and plant architecture throughout development.

### KASII overexpression remodels membrane lipid composition

To determine whether the chloroplast disorganization observed in KASIIox plants was accompanied by altered membrane composition, we performed comprehensive lipidomic profiling of rosette tissues from control, KASIIox, and SSI2ox lines grown under long‐day growth conditions (Figure 2; Table S2).

**Figure 2:**
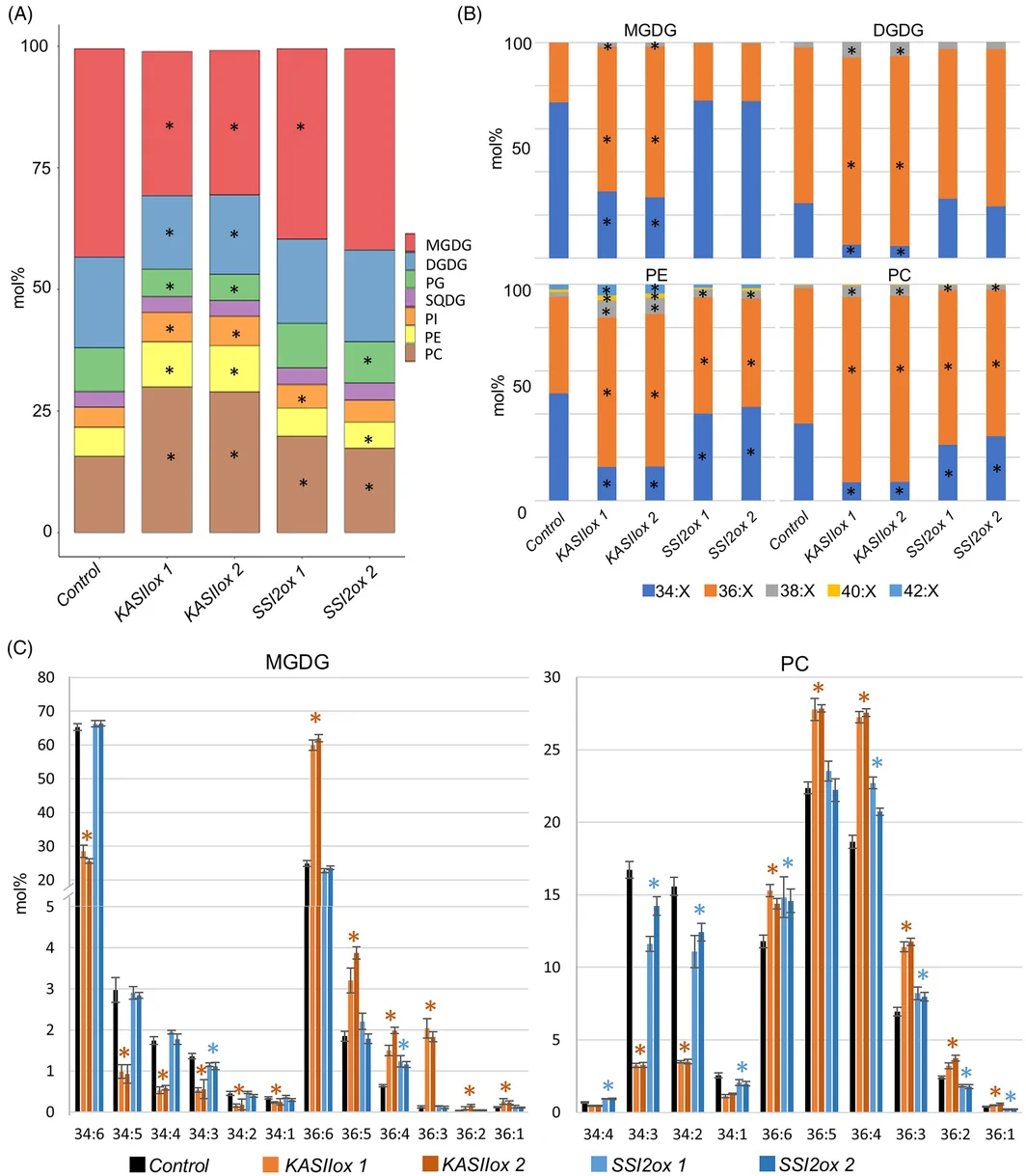
Plastidial fatty‐acid condensation reshapes membrane lipid composition and acyl‐chain distribution. (A) Relative mol% abundance of major membrane lipid classes in rosette tissue from *Arabidopsis thaliana* control, KASII overexpression (KASIIox 1, KASIIox 2), and SSI2 overexpression (SSI2ox 1, SSI2ox 2) lines. Plastidial membrane lipids include monogalactosyldiacylglycerol (MGDG), digalactosyldiacylglycerol (DGDG), phosphatidylglycerol (PG), and sulfoquinovosyldiacylglycerol (SQDG), whereas extraplastidial phospholipids include phosphatidylcholine (PC), phosphatidylethanolamine (PE), and phosphatidylinositol (PI). Asterisks denote statistically significant differences relative to controls (*n =* 5; Welch's *t*‐test with Benjamini–Hochberg FDR correction, adjusted *P* ≤ 0.05). (B) Distribution of fatty‐acid chain‐length classes within individual lipid species (MGDG, DGDG, PE, and PC) across the indicated genotypes. Chain‐length classes are defined by the combined carbon number of both acyl chains per lipid molecule, independent of unsaturation. Asterisks indicate statistically significant differences relative to controls (*n =* 5; Welch's *t*‐test with Benjamini–Hochberg FDR correction, adjusted *P* ≤ 0.05). (C) Detailed acyl‐chain profiles of representative plastidial (MGDG) and extraplastidial (PC) lipid classes in control, KASIIox, and SSI2ox lines. Data represents mean ± SD. Color coded asterisks (KASIIox‐ orange; SSI2ox‐ blue) indicate statistically significant differences in both independent transformants relative to controls (*n =* 5; Welch's *t*‐test with Benjamini–Hochberg FDR correction, adjusted *P* ≤ 0.05). Full statistical analysis for all lipid classes in Table S2.

KASII overexpression caused pronounced remodeling of membrane lipid composition (Figure 2A,B). Compared with controls, KASIIox lines exhibited marked reductions in the plastid‐enriched galactolipids MGDG and DGDG, accompanied by increased abundance of the extraplastidial phospholipids PC, PE, and PI. These changes were evident not only in relative lipid abundance (mol%; Figure 2A) but also in absolute quantification, with PC levels increasing from 33 nmol mg^−1^ dry weight in controls to 45–47 nmol mg^−1^ dry weight in KASIIox lines (Table S2). This reciprocal shift indicates redistribution of glycerolipid assembly away from plastidial galactolipids toward ER‐associated phospholipid synthesis. By contrast, SSI2 overexpression produced only modest changes in lipid class abundance, highlighting distinct functional consequences of plastidial fatty‐acid condensation versus desaturation activities.

We next examined whether these lipidomic changes were accompanied by altered acyl‐chain composition. KASII overexpression shifted both plastidial and extraplastidial membrane lipids toward longer‐chain species, with strong enrichment of 36‐carbon molecular species specifically in MGDG and PC (Figure 2C), consistent with increased incorporation of C18 fatty acids generated by KASII‐mediated condensation. In contrast, SSI2ox lines remained largely similar to controls, exhibiting only modest increases in specific elongated acyl‐chain species within the ER phospholipids PC and PE. These findings indicate that elevated plastidial acyl‐chain output extends beyond the chloroplast to broadly remodel cellular membrane composition.

Collectively, these findings demonstrate that sustained KASII overexpression constitutively reprograms both lipid class abundance and acyl‐chain composition. Coordinated depletion of plastidial galactolipids, expansion of ER phospholipids, and enrichment of longer‐chain lipid species indicate rebalancing of membrane biogenesis between plastidial and ER‐associated glycerolipid pathways. This compartment‐spanning remodeling of membrane composition provides a mechanistic framework for the chloroplast ultrastructural defects and developmental phenotypes observed in KASIIox plants.

### Plastidial fatty‐acid condensation disrupts chloroplast ultrastructure and mesophyll organization

To determine whether lipid remodeling associated with elevated plastidial fatty‐acid condensation influences tissue organization, we examined the anatomy of the first true leaves in KASIIox and SSI2ox seedlings using a modified pseudo‐Schiff propidium iodide staining (mPS‐PI) to visualize whole fixed tissues by confocal microscopy. Consistent with their severe leaf developmental phenotype (Figures 1A, 3A; Figure S3), KASIIox leaves displayed profound alterations in mesophyll organization compared with control and SSI2ox plants (Figure S3B). In control seedlings, mesophyll cells formed a compact and continuous tissue with regular cellular geometry and close association between neighboring cells. By contrast, KASIIox leaves exhibited enlarged and irregularly shaped mesophyll cells, extensive intercellular spaces, and a loss of coordinated tissue organization. These defects were observed in two independent KASIIox lines and were particularly evident at higher magnification, where normal mesophyll packing was largely absent (Figure 3B).

**Figure 3:**
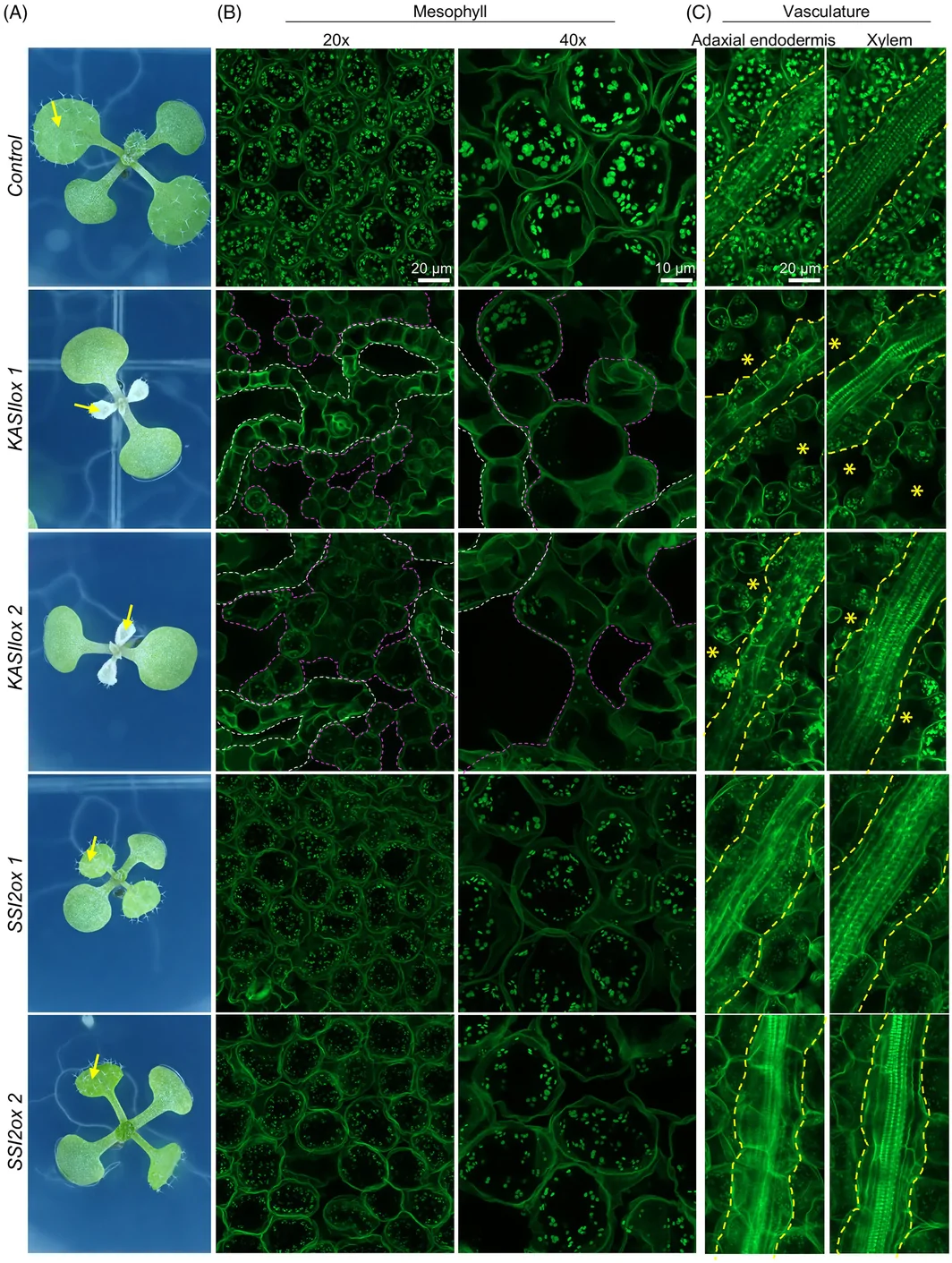
Plastidial fatty‐acid condensation disrupts mesophyll organization and mesophyll–vascular connectivity. (A) Representative images of 10‐day‐old *Arabidopsis thaliana* seedlings from control, KASII overexpression (KASIIox 1, KASIIox 2), and SSI2 overexpression (SSI2ox 1, SSI2ox 2) lines. Yellow arrows indicate the first true leaves used for tissue imaging and anatomical analyses. (B) Confocal imaging of mesophyll organization in first true leaves stained with mPS‐PI to visualize cell walls and starch granules. Images were acquired at 20× and 40× magnification. White dashed lines delineate epidermal cell boundaries, whereas magenta dashed lines outline mesophyll cells. KASII overexpression markedly alters mesophyll architecture, producing enlarged intercellular spaces, irregular cell geometry, and disrupted tissue organization. By contrast, SSI2 overexpression largely preserves mesophyll organization comparable to controls, although starch granule accumulation appears reduced. A total of 10 leaves per experimental group were sampled from two independent experiments; representative max intensity projections are shown. (C) Confocal optical sections of vascular‐associated regions within first true leaves. Yellow dashed lines outline vascular boundaries. In KASII overexpression lines, mesophyll cells become spatially separated from vascular tissues, generating pronounced interstitial air spaces (asterisks) and compromised mesophyll–vascular connectivity. In contrast, SSI2 overexpression lines maintain vascular organization and tissue connectivity similar to controls. Scale bars: 20 μm (20× images) and 10 μm (40× images).

The anatomical defects extended beyond the mesophyll to regions surrounding the vasculature. In control and SSI2ox leaves, mesophyll cells were closely associated with vascular tissues, maintaining continuous tissue organization from the vascular bundle into the surrounding photosynthetic tissue (Figure 3C). In contrast, KASIIox leaves displayed pronounced gaps between vascular tissues and adjacent mesophyll cells, generating large interstitial air spaces and disrupting normal mesophyll–vascular connectivity (Figure 3C, asterisks). These defects suggest a failure to maintain proper tissue cohesion and spatial organization during leaf development. Notably, vascular morphology itself appeared largely unaffected, indicating that elevated KASII activity primarily affects the organization and integration of surrounding mesophyll tissues rather than vascular differentiation per se.

In contrast to KASII overexpression, SSI2ox leaves retained mesophyll architecture and mesophyll–vascular connectivity comparable to controls, despite exhibiting a modest reduction in starch granule accumulation (Figure 3B,C). Together, these findings demonstrate that enhanced plastidial fatty‐acid condensation, but not fatty‐acid desaturation, profoundly alters leaf tissue architecture, disrupting both mesophyll organization and the physical continuity between photosynthetic and vascular tissues. These observations suggest that membrane lipid composition generated through KASII‐dependent acyl‐chain remodeling is an important feature associated with higher‐order tissue patterning during leaf development.

### Plastidial fatty‐acid condensation uncouples light‐dependent morphogenesis from adaptive low‐light responses

Because the developmental defects associated with KASII overexpression were most pronounced in photosynthetic tissues, we next asked whether these phenotypes depend on light and whether altered plastidial fatty‐acid metabolism influences adaptive growth responses to changes in light availability. Comparison of seedlings grown under standard light or in complete darkness revealed that the effects of KASII overexpression were strongly light dependent (Figure 4A,B). Under standard light conditions, KASIIox cotyledons were enlarged and displayed abnormal cellular organization characterized by enlarged and irregular epidermal cells together with increased chloroplast size (Figure 4C). In contrast, dark‐grown cotyledons exhibited cellular organization and plastid morphology largely comparable to controls, indicating that the developmental consequences of elevated KASII activity require light‐dependent processes. SSI2ox seedlings remained similar to controls under both growth conditions (Figure 4C).

**Figure 4:**
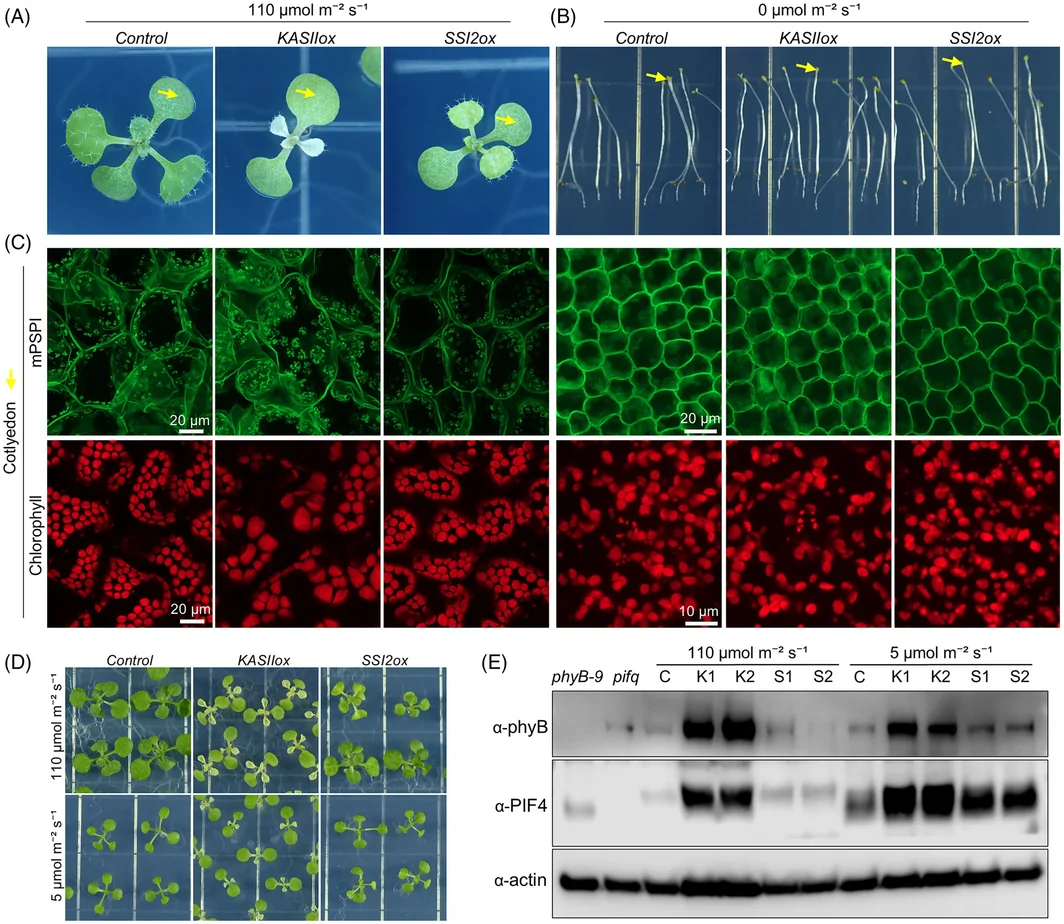
Plastidial fatty‐acid condensation uncouples light‐dependent leaf morphogenesis from adaptive low‐light responses. (A) Representative images of *Arabidopsis thaliana* control, KASII overexpression (KASIIox), and SSI2 overexpression (SSI2ox) seedlings grown under (110 μmol m^−2^ s^−1^) or in darkness (0 μmol m^−2^ s^−1^; B). Yellow arrows indicate cotyledons used for cellular imaging and anatomical analyses shown in (C). KASII overexpression alters cotyledon development under light conditions but not in darkness, indicating a light‐dependent phenotype. (C) Confocal imaging of mesophyll organization and chloroplast morphology in cotyledons of light and dark grown plants. Cell walls were visualized by mPS‐PI staining (green), and chloroplasts by chlorophyll autofluorescence (red). Scale bars: 20 μm; 10 μm for chlorophyll in dark grown seedlings. A total of 10 leaves per experimental group were sampled from two independent experiments; representative max intensity projections are shown. (D) Representative rosette phenotypes of 12‐day‐old control, KASIIox, and SSI2ox seedlings grown under standard light (110 μmol m^−2^ s^−1^) for 7 days, then transferred to low light (5 μmol m^−2^ s^−1^) for 5 days. Control and SSI2ox plants exhibit the characteristic low‐light response of petiole elongation, whereas KASIIox plants fail to undergo this adaptive developmental transition and maintain a compact rosette architecture. (E) Immunoblot analysis of phyB and PIF4 protein abundance in seedlings grown under standard light (110 μmol m^−2^ s^−1^) or transferred to low light (5 μmol m^−2^ s^−1^) for 5 days. C, control; K1 and K2, independent KASII overexpression lines; S1 and S2, independent SSI2 overexpression lines; with *phyB‐9* and *pifq* mutants included as controls for phyB and PIF4 blots respectively. Actin served as a loading control. Three biological replicates were assayed, with a representative blot of 1 biological replicate shown; the second and third found in Figure S6 with quantification and statistical analysis provided.

We next examined whether altered plastidial fatty‐acid condensation affects developmental acclimation to reduced light availability. Seven‐ day‐old seedlings grown under standard light (110 μmol m^−2^ s^−1^) were transferred to low light (5 μmol m^−2^ s^−1^) for an additional 5 days. Under low light all genotypes displayed reduced leaf sizes consistent with the lower light intensity (Figure 4D; Figure S5). Additionally, control and SSI2ox plants exhibited the characteristic low‐light response of increased petiole elongation relative to leaf size. (Figure 4D; Figure S5). In contrast, KASIIox plants failed to exhibit this response and retained a compact rosette architecture, suggesting that KASII overexpression compromises developmental acclimation to low‐light conditions.

To examine whether the altered low‐light growth response of KASIIox plants was accompanied by changes in light‐responsive regulators, we assessed the protein abundance of phyB and PIF4. Under standard light, KASIIox lines showed higher levels of both phyB and PIF4 relative to controls (Figure 4E; Figure S6). Under low light, PIF4 abundance remained higher in KASIIox plants than in control and SSI2ox lines, whereas differences in phyB abundance were less apparent. Notably, the elevated PIF4 abundance in KASIIox plants was not accompanied by the characteristic low‐light petiole elongation observed in control and SSI2ox plants. Thus, the altered developmental response of KASIIox plants is associated with changes in phyB and PIF4 protein abundance, although the relationship between these changes and the impaired low‐light growth response remains unresolved.

### Plastidial fatty‐acid condensation disrupts seed coat mucilage deposition without detectable alterations in embryonic leaf patterning

Because KASII overexpression caused pronounced postembryonic defects in leaf architecture, we next asked whether elevated plastidial fatty‐acid condensation is also associated with alterations in embryonic cotyledon organization or other seed morphological traits. mPS‐PI staining and confocal imaging revealed no obvious differences in cotyledon epidermal and mesophyll organization in KASIIox and SSI2ox embryos relative to controls (Figure 5A). Thus, within the embryonic tissues examined, altered plastidial acyl‐chain output was not associated with detectable changes in cotyledon epidermal or mesophyll organization.

**Figure 5:**
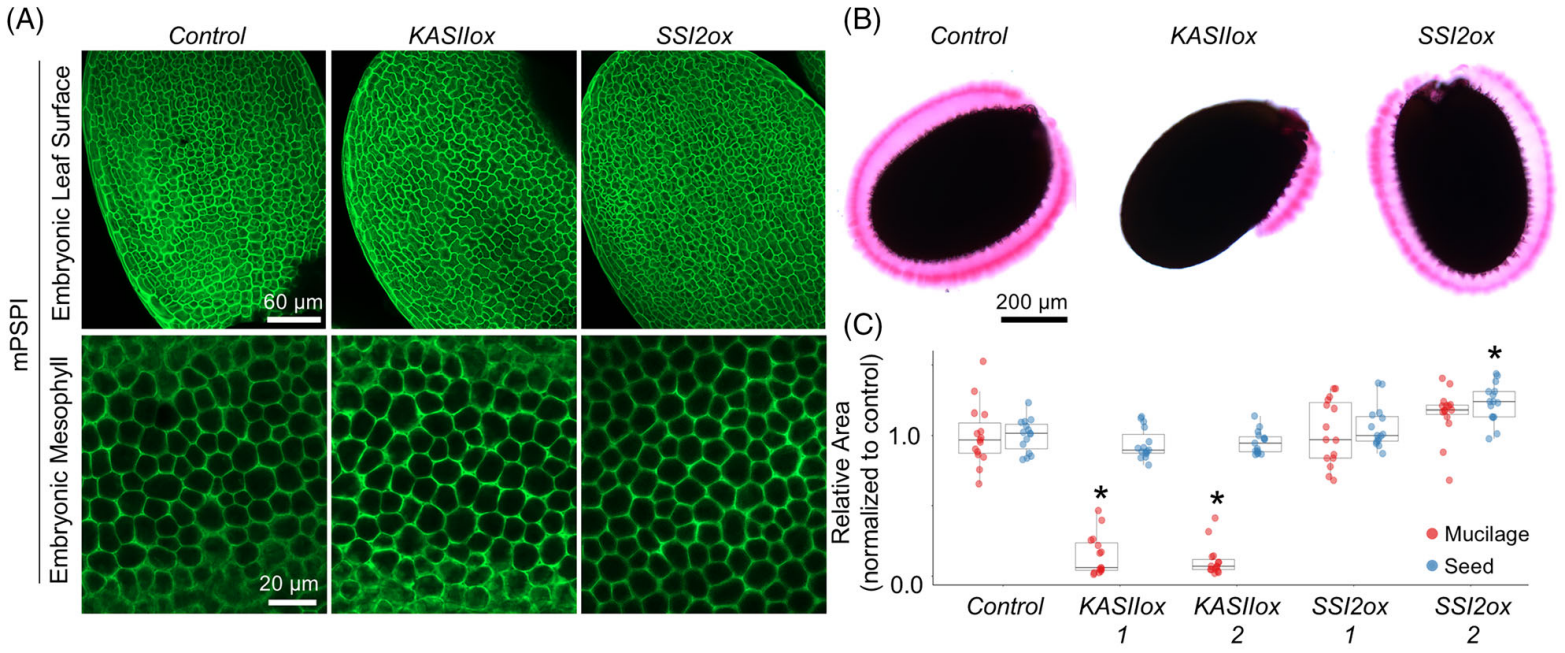
Plastidial fatty‐acid condensation disrupts seed coat mucilage, but not embryonic tissue organization. (A) Confocal imaging of embryonic cotyledon surface and mesophyll organization in developing embryos from control, KASII overexpression (KASIIox), and SSI2 overexpression (SSI2ox) lines stained with mPS‐PI to visualize cell walls. Neither KASII nor SSI2 overexpression substantially alters embryonic cellular organization or tissue patterning within cotyledon epidermal and mesophyll layers. Scale bars: 60 μm (top panels) and 20 μm (bottom panels). A total of 10 embryos from each experimental group were sampled; representative max intensity projections are shown. (B) Mature seeds were imbibed for 24 h at 4°C then stained with ruthenium red to visualize extruded seed coat mucilage. Scale bar: 200 μm. (C) Quantification of relative mucilage area and seed area (used as a measure of seed size) normalized to controls. KASII overexpression significantly reduces mucilage extrusion relative to controls, whereas SSI2 overexpression has comparatively minor effects. Box plot shows median, interquartile range, and points as individual biological replicates; asterisks indicate statistically significant differences relative to control (*n* = 15; two‐way ANOVA, *P* ≤ 0.05).

Despite the largely normal embryonic organization, all KASIIox seeds exhibited a striking reduction in adherent seed coat mucilage following ruthenium red staining, whereas SSI2ox seeds largely resembled controls (Figure 5B,C). Quantification confirmed a substantial decrease in mucilage area specifically in KASIIox seeds without affecting overall seed size. These findings suggest that altered plastidial acyl‐chain remodeling selectively impacts postembryonic architectural programs such as leaf development and seed coat formation rather than broadly disrupting cotyledon formation and development during embryogenesis.

### Altered gibberellin responsiveness links plastidial acyl‐chain remodeling to developmental organization

Given the established roles of gibberellins (GA) in tissue expansion, epidermal differentiation, leaf morphogenesis (Cheminant et al., 2011; de Lucas et al., 2008; Feng et al., 2008; Schwechheimer, 2011) and the recently established requirement for gibberellin in pectin formation within seed coat mucilage, disruption of which produces phenotypes resembling the reduced mucilage observed in KASIIox seeds (Xu et al., 2025), we next examined whether the architectural defects associated with KASII overexpression were responsive to altered GA status. Seedlings were therefore germinated and grown under standard conditions for 4 days, then sprayed with GA_3_ or the GA biosynthesis inhibitor paclobutrazol (PAC) (Figure 6; Figure S7).

**Figure 6:**
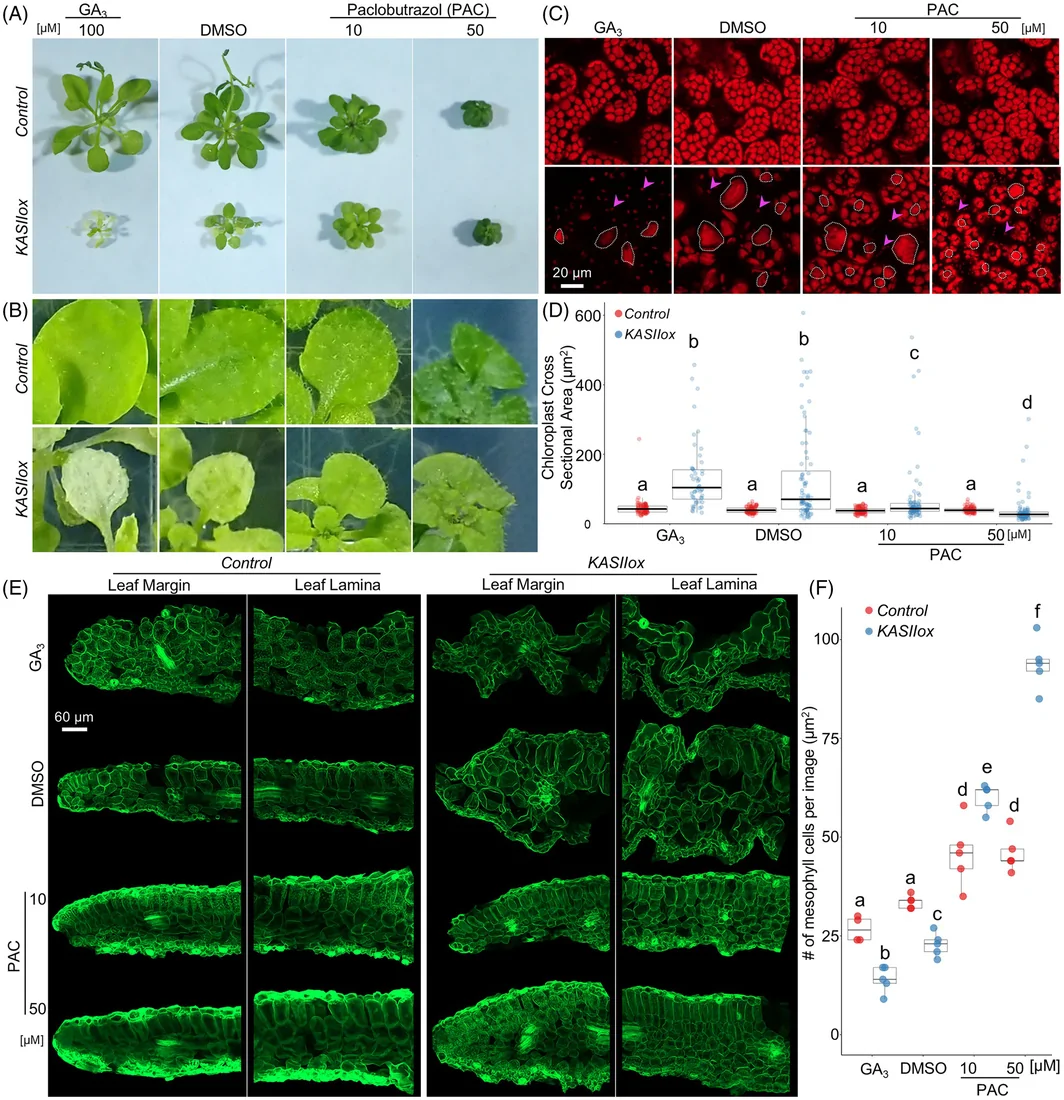
Gibberellin perturbation uncovers a role for plastidial acyl‐chain remodeling in chloroplast organization and mesophyll patterning. (A) Representative 35‐day‐old rosette phenotypes of control and KASIIox plants grown in sterile culture conditions sprayed with sterile GA_3_ (100 μM), paclobutrazol (PAC; 10 or 50 μM), or DMSO control 4 days after germination under long day conditions. (B) Representative images of first true leaves from 21‐day‐old seedlings grown under the treatments described in (A). Under DMSO conditions, KASIIox leaves display reduced expansion, irregular lamina morphology, and chlorosis compared with controls. These defects are further exacerbated by GA_3_ treatment, whereas PAC partially restores leaf shape, tissue organization, and overall leaf morphology. (C) Chlorophyll autofluorescence imaging of mesophyll chloroplasts in 21‐day‐old. Compared with controls, KASIIox leaves exhibit pronounced alterations in chloroplast size and abundance under DMSO conditions. These defects are further enhanced by GA_3_ treatment, whereas PAC promotes a reduction in chloroplast size within mesophyll tissues of KASIIox plants. Scale bar = 20 μm. A total of 10 leaves per experimental group were sampled from two independent experiments, and representative max intensity projections are shown. Mesophyll chloroplasts in KASIIox leaves were identified by cell location below adaxial epidermis as determined from concurrently collected transmitted light images (a representative range of chloroplast sizes are outlined in dashed white lines). Representative, smaller chloroplasts found in epidermal cells are identified by magenta arrows. (D) Quantification of chloroplast size from images shown in (C). From max intensity projections, cross‐sectional area from twenty mesophyll chloroplasts from 4 randomly selected images was measured (*n* = 80). The number of sampled mesophyll chloroplasts in GA_3_ treated KASIIox plants is reduced due to lower numbers of mesophyll chloroplasts per image (*n* = 51). Box plot shows median, interquartile range, and points as individual chloroplast areas; different letters denote statistically significant differences (two‐way ANOVA, *P* ≤ 0.05). (E) First true leaves of 21‐day‐old plants were fixed, stained with mPS‐PI, and cut to produce adaxial to abaxial transverse cross sections midway between the leaf apex and base. The leaf margin and lamina to the left or right of the midvein were then imaged by confocal microscopy (Z‐series; 5–10 μm at 1 μm intervals). Scale bar = 60 μm. A total of 10 leaves per experimental group were sampled from two independent experiments; representative max intensity projections are shown. Backgrounds from original images were cropped out to allow for rotation and presentation of the vertical cross sections in the same adaxial‐abaxial orientation over a black background. Brightness of entire PAC‐treated control images was enhanced to better show mesophyll cell boundaries. (F) Quantification of the number of mesophyll cells per image of 5 randomly selected leaf centers imaged in (E). Box plot shows median, interquartile range, and points as the quantified value from an individual leaf; different letters denote statistically significant differences (*n* = 5; two‐way ANOVA, *P* ≤ 0.05).

When grown to maturity (35 DAG), KASIIox plants exhibited dwarfing, partially chlorosis, phenotype that became more pronounced following exogenous GA_3_ treatment. Whereas GA_3_ promoted substantial rosette expansion in control plants, GA_3_‐treated KASIIox plants remained small and developed pronounced chlorosis, with some rosettes appearing nearly albino (Figure 6A; Figure S7). In contrast, treatment with 10 μM PAC reduced rosette size in control plants but increased rosette size in KASIIox plants relative to their respective DMSO‐treated control plants. At higher PAC concentrations, the morphological differences between control and KASIIox plants became less apparent.

Examination of developing leaves from 21‐day‐old seedlings further revealed that under DMSO control conditions, KASIIox leaves exhibited reduced width, irregular lamina morphology, and chlorosis (Figure S7). These phenotypes became more pronounced following GA_3_ treatment. Conversely, PAC treatment reduced leaf size in control plants but partially ameliorated the abnormal lamina morphology of KASIIox leaves, with comparatively little effect on their overall leaf size (Figure 6B; Figure S7).

To determine whether these phenotypic responses reflected altered cellular organization, we examined chloroplast distribution and mesophyll architecture. Under DMSO conditions, KASIIox leaves exhibited marked abnormalities in chloroplast size relative to controls (Figure 6C,D). In contrast to control chloroplasts, whose size was largely unaffected by either GA_3_ or PAC treatment, KASIIox chloroplasts responded strongly to PAC, decreasing in size to near‐control levels at 10 μM. Although GA_3_ did not further increase chloroplast size in KASIIox plants, it markedly reduced the abundance of mesophyll chloroplasts within the imaging field, while the smaller epidermal chloroplasts remained evident across all treatments. Consistent with these observations, adaxial to abaxial transverse cross sections revealed extensive disruption of mesophyll architecture in KASIIox leaves (Figure 6E,F; Figure S8). While GA_3_‐treated control leaves showed a trend toward fewer, larger mesophyll cells and 10 μM PAC‐treated leaves contained significantly more mesophyll cells, control plants maintained well‐defined palisade and spongy mesophyll layers across all treatments. In contrast, KASIIox leaves exhibited irregular cell morphology, reduced expansion of palisade mesophyll cells, loss of tissue continuity, and extensive intercellular spaces under DMSO conditions (Figure 6F; Figure S8). These defects were further aggravated by GA_3_ treatment, producing poorly differentiated mesophyll tissues and hollow leaf structures largely devoid of internal mesophyll cells. By contrast, inhibition of GA biosynthesis with 10 μM PAC largely restored mesophyll organization in KASIIox leaves, increasing cellular density, improving tissue continuity, and returning cell size to near‐control levels (Figure 6F; Figure S8). Notably, higher PAC concentrations produced a further increase in mesophyll cell number, a response not observed in control leaves. Together, these findings position plastidial acyl‐chain composition as an important feature associated with gibberellin‐responsive developmental competence.

We then repeated chloroplast and cellular morphology analyses, comparing the responses of the control and SSI2ox plants to GA perturbation (Figure S9). While quantitative analysis found that SSI2ox displayed slightly smaller chloroplasts (Figure S9) and palisade cell size (Figure S9), overall mesophyll architecture and the relative responses to the GA and PAC were largely unchanged compared to the control.

In summary, exogenous GA intensified, whereas GA inhibition partially alleviated the morphological defects of KASIIox tissues, revealing differential responses of KASIIox plants to altered GA status. Together with the pronounced reduction in seed coat mucilage and the absence of detectable alterations in the embryonic tissues examined, these findings further associate altered plastidial fatty‐acid condensation with specific aspects of postembryonic leaf development.

### KASII overexpression alters GA‐associated transcriptional responses

To further examine the relationship between KASII overexpression and GA‐associated responses, we assessed GA abundance, RGA1 protein levels, and the expression of GA‐responsive genes (Figure 7A). Using targeted LC–MS/MS, several phytohormones were detected, including ABA, IAA, ICA, JA, JA‐Ile, SA, and trans‐zeatin; however, bioactive GAs and GA intermediates were below the detection limit under our experimental conditions (Figure S10).

**Figure 7:**
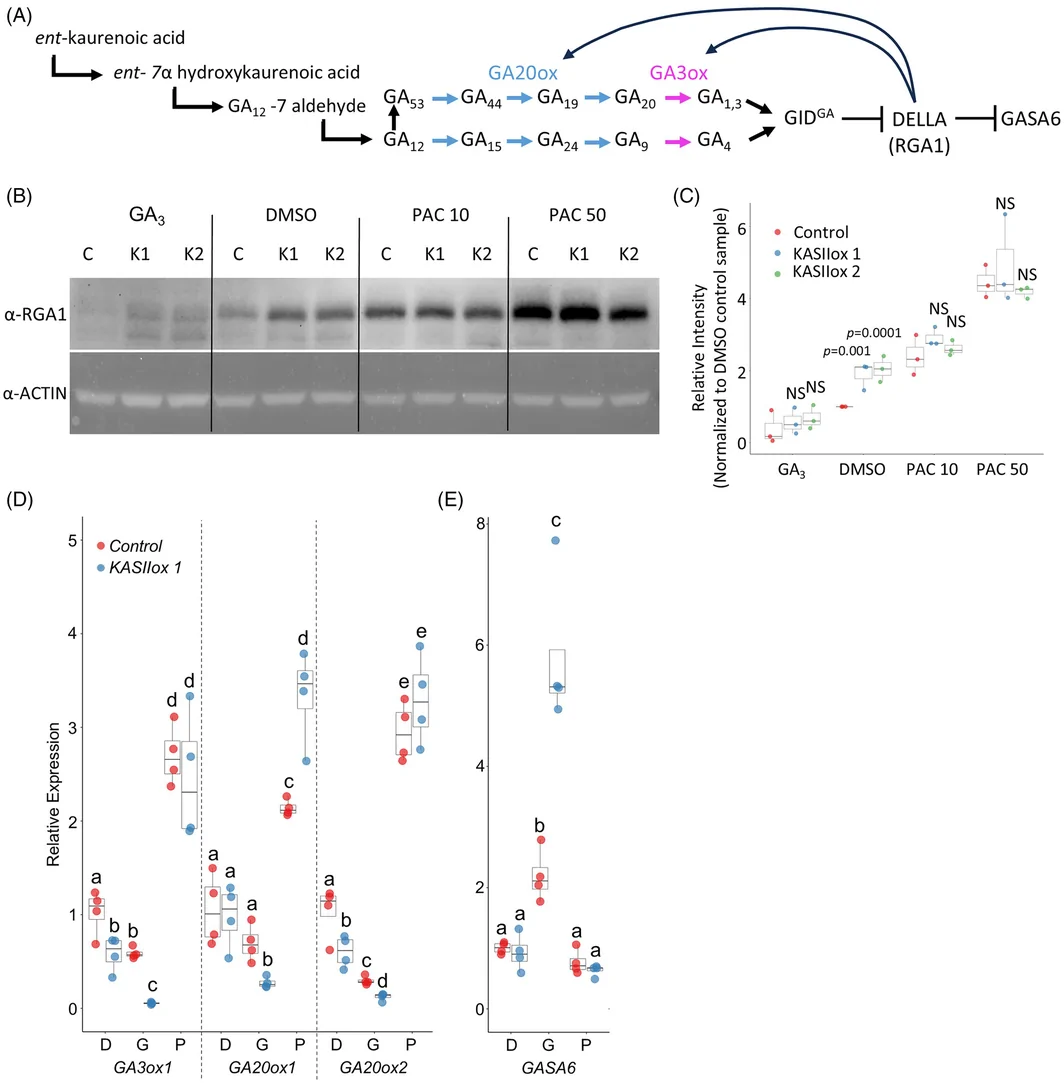
Plastidial fatty‐acid condensation enhances GA‐associated transcriptional responses. (A) Schematic of GA biosynthesis pathway occurring at the endoplasmic reticulum leading to the production of bioactive GA_1_, GA_3_, and GA_4_ followed by an overview of subsequent cellular signaling events assayed in this work, including RGA1 protein levels, which can be negatively affected by GID‐GA‐dependent degradation, and downstream transcriptional regulation of GA3ox1, GA20ox1, GA20ox2, and GASA6 by RGA1/DELLA. (B) Representatives immunoblot of RGA1 (~63 kDA in size) and loading control actin (~37 kDA in size) from 21‐day‐old, excised shoots treated with 100 μm GA_3_, DMSO vehicle control, 10 μm or 50 μm paclobutrazol at 4 days and 14 days after germination. C = control, K1 = KASIIox 1, K2 = KASIIox 2. Full blots provided for all three biological replicates in Figure S11. (C) Quantification of RGA1 band intensity from all three biological replicates normalized to intensity of control line treated with DMSO vehicle in Figure 7B and Figure S11. Box plot shows median, interquartile range, and points as the quantified and normalized value from an individual band; NS denotes KASIIox experimental groups which are not significantly different from the control for each treatment. Statistically significant differences from control denoted by displayed *P*‐values. (*n* = 3; two‐way ANOVA). (D) Relative transcript abundance of the GA‐biosynthetic genes (*GA3ox1, GA20ox1, GA20ox2*) measured by RT–qPCR in the indicated genotypes and treatments (treatment abbreviations: D‐ DMSO vehicle, G‐ 100 μm GA_3_, P‐ 10 μm PAC). Data is shown relative to mock‐treated controls. Box plots indicate median and interquartile range, with points as individual biological replicates representing measurements from RNA sourced from independent pools of shoot tissue; different letters denote statistically significant differences tested individually for each transcript. (*n* = 4; two‐way ANOVA, *P* ≤ 0.05). (E) Relative transcript abundance of the Gibberellic Acid Stimulated Arabidopsis 6 (*GASA6*) gene measured by RT–qPCR in the indicated genotypes and treatments (treatment abbreviations: D‐ DMSO vehicle, G‐ 100 μm GA_3_, P‐ 10 μm PAC). Data is shown relative to mock‐treated controls. Box plots indicate median and interquartile range, with points as individual biological replicates representing measurements from RNA sourced from independent pools of shoot tissue; different letters denote statistically significant differences. (*n* = 4; two‐way ANOVA, *P* ≤ 0.05).

We next examined RGA1, a DELLA protein whose abundance is regulated by GA‐dependent proteasomal degradation actuated by the GID1 receptor complex (Nakajima et al., 2006; Ueguchi‐Tanaka et al., 2005). In control plants, exogenous GA reduced RGA1 abundance, whereas increasing concentrations of the GA biosynthesis inhibitor PAC increased RGA1 levels, confirming the expected responses to these treatments (Figure 7B,C; Figure S11). Although RGA1 appeared slightly elevated in DMSO‐treated KASIIox plants, normalization to actin revealed no significant differences between KASIIox and control plants under any condition tested (Figure S11).

Because DELLA activity can also be modulated independently of changes in protein abundance (Blanco‐Touriñán et al., 2020; Fernandes et al., 2024; Huang et al., 2024; Zentella et al., 2016; Zentella et al., 2017), we examined GA‐responsive transcripts downstream of this pathway. In control plants, GA treatment reduced GA3ox1, GA20ox1, and GA20ox2 transcript abundance twofold, consistent with GA‐mediated feedback regulation (Figure 7D) (Hu et al., 2018; Zhong et al., 2015). Under DMSO conditions, KASIIox plants exhibited lower basal levels of GA3ox1 and GA20ox2 transcripts than controls. Exogenous GA further reduced GA3ox1 transcript abundance in KASIIox plants, while GASA6 showed a greater GA‐induced increase in KASIIox than in control plants (fivefold versus twofold; Figure 7D,E). In contrast, PAC treatment increased GA‐biosynthetic transcripts to similar levels in KASIIox and control plants.

Together, these results reveal altered GA‐associated transcriptional responses in KASIIox plants despite no detectable difference in RGA1 abundance. The convergence of transcript responses between KASIIox and control plants following PAC treatment is also consistent with the partial amelioration of KASIIox leaf phenotypes under GA biosynthesis inhibition. These findings establish an association between KASII overexpression and altered GA‐responsive transcription but do not distinguish whether this response results from changes in GA abundance, signaling activity, or downstream regulatory processes.

## DISCUSSION

The discovery that the stress‐related plastidial metabolite MEcPP may associate with enzymes of fatty‐acid biosynthesis provided the conceptual foundation for examining how perturbation of plastidial acyl‐chain metabolism relates to organelle organization and plant development. Although the present study does not establish direct MEcPP binding to KASII or SSI2 or further define the role of MEcPP in regulating these enzymes, this initial observation prompted us to investigate the biological consequences of sustained alteration of two sequential steps in plastidial fatty‐acid metabolism: acyl‐chain condensation by KASII and desaturation by SSI2.

Fatty‐acid metabolism is generally viewed through its essential roles in membrane biogenesis, energy storage, and the generation of lipid‐derived signals. Our findings suggest that perturbation of a specific step in plastidial fatty‐acid metabolism can generate a cellular state in which organelle organization, tissue differentiation, and adaptive growth are altered together. Importantly, these consequences were strongly associated with sustained elevation of KASII but were much less evident following elevation of SSI2, despite the sequential functions of these enzymes in plastidial fatty‐acid metabolism. KASII overexpression was associated with extensive remodeling of membrane lipid composition, altered chloroplast and tissue organization, and impaired developmental responses to changing light and chemical perturbation of GA status, whereas SSI2 overexpression produced more limited effects. This distinction argues against the developmental phenotypes being a general consequence of increased fatty‐acid enzyme abundance and instead points to KASII‐dependent acyl‐chain remodeling as an important feature associated with developmental outcome.

The essential function of KASII presents an inherent challenge for investigating this relationship through conventional loss‐of‐function approaches. Complete loss of KASII/FAB1 function is embryo lethal, and strong suppression of KASII similarly compromises embryo development (Pidkowich et al., 2007). In addition to the embryo lethality of the *fab1‐2* complete loss‐of‐function allele, strong KASII/FAB1 hairpin‐RNAi also caused embryo abortion (Hakozaki et al., 2008), making it difficult to distinguish postembryonic consequences of altered KASII‐dependent metabolism from its fundamental requirement during fatty‐acid biosynthesis and embryogenesis. Partial‐loss‐of‐function alleles have been informative but likewise produce less substantial alterations in fatty‐acid composition and have WT‐like phenotypes when grown in standard 22°C growth conditions (Carlsson et al., 2002; Jinpeng et al., 2015; Wu et al., 1997). Sustained KASII elevation therefore provided a complementary means to perturb this metabolic step without eliminating its essential function and allowed us to examine how a shift in KASII‐dependent acyl‐chain metabolism is associated with subsequent membrane, cellular, and developmental outcomes. Nevertheless, because overexpression represents an experimental perturbation, these findings should not be interpreted as demonstrating the consequences of physiological fluctuations in endogenous KASII activity.

### KASII‐dependent acyl‐chain remodeling and cellular membrane composition

Lipid profiling provided a biochemical context for the striking developmental difference between KASIIox and SSI2ox plants. KASII overexpression was associated with a pronounced redistribution from plastid‐enriched galactolipids toward extraplastidial phospholipids and enrichment of longer‐chain molecular species, whereas SSI2 overexpression produced comparatively modest changes. These findings are particularly informative because KASII and SSI2 act sequentially but modify different properties of the fatty‐acid pool, with KASII extending C16 acyl chains to C18, whereas SSI2 subsequently desaturates C18.

The contrasting phenotypic outcomes raise the possibility that altering acyl‐chain elongation has consequences that are distinct from desaturation. Given the requirement for C16 in plastidial lipid synthesis (Li‐Beisson et al., 2013), the balance between C16‐ and C18‐derived acyl chains may influence how fatty acids are partitioned among plastidial and extraplastidial membrane systems. The observed decrease in plastid‐enriched galactolipids and expansion of ER‐derived phospholipids (Figure 2) is consistent with such a redistribution. However, whether these changes arise directly from altered substrate availability, compensatory lipid homeostasis, altered exchange between plastids and the ER, or secondary consequences of impaired development remain unresolved.

This distinction has potentially broader significance because membrane identity depends not only on the amount of lipid produced but also on lipid class, acyl‐chain composition, and the physical properties that emerge from their combination. KASIIox chloroplasts displayed altered thylakoid organization in parallel with changes in lipid composition, suggesting that chloroplast architecture is particularly sensitive to this metabolic imbalance. MGDG and DGDG have important structural roles in photosynthetic membranes, where their abundance and molecular composition contribute to membrane packing, curvature, and protein–lipid interactions (Dormann & Benning, 2002; Kobayashi, 2016). Altering both their abundance and acyl‐chain composition could therefore change the membrane environment required for normal thylakoid organization. In this context, the chloroplast phenotype may reflect consequences of altered membrane homeostasis rather than a direct action of KASII itself. Establishing causality will require determining whether restoration of specific lipid classes or acyl‐chain distributions is sufficient to restore chloroplast architecture.

### From organelle metabolism to tissue organization

The consequences associated with KASII overexpression extended beyond chloroplast architecture to the organization of the leaf itself. KASIIox leaves displayed disrupted mesophyll packing, reduced palisade organization, and increased intercellular spaces, whereas tissue organization in SSI2ox remained more similar to controls. These observations suggest that the metabolic state associated with KASII elevation may become consequential at levels of biological organization beyond the individual organelle.

This connection is biologically plausible because leaf morphogenesis requires the coordinated execution of several membrane‐intensive processes. Cell expansion, membrane proliferation, chloroplast differentiation, intercellular communication, and establishment of photosynthetic tissue architecture must proceed together as leaves develop (Kozuka et al., 2011; Terashima et al., 2006). The reduction in adherent seed coat mucilage in KASIIox lines broadens this perspective beyond photosynthetic tissues. Mucilage production and subsequent extrusion during seed imbibition require coordinated polysaccharide synthesis, membrane trafficking, secretion, and extracellular matrix assembly within specialized epidermal cells (Arsovski et al., 2010; Western et al., 2000). Because overall seed size was not substantially altered, the mucilage phenotype is unlikely to represent a generalized disruption in seed development. Instead, the mucilage phenotype could arise indirectly from related developmental changes, such as those disrupted by altered GA‐associated signaling (Figure 7) (Xu et al., 2025). Alternatively, it may be possible that KASIIox plastid fatty‐acid metabolism influences membrane‐dependent cellular machinery which may require specific lipid compositions for proper execution of complex macromolecule synthesis and delivery. Rather than implying that KASII directs mucilage production, or mesophyll and palisade identity, our findings suggest that an altered metabolic and membrane environment may possibly constrain the normal cellular processes required for these tissues to acquire and maintain their standard architecture by affecting the capacity of differentiating cells to coordinate growth with organelle biogenesis and tissue organization.

The relatively unaffected organization of KASIIox embryonic cotyledons provides an important qualification to this interpretation. KASII overexpression does not appear to prevent tissue specification at this early stage. Instead, the strongest abnormalities become evident during postembryonic growth and differentiation of leaf tissues. This distinction suggests that the KASII‐associated metabolic state may affect developmental plasticity, the capacity of an already specified tissue to execute and adjust its developmental program as growth proceeds, more strongly than initial cell‐fate determination. Such an interpretation provides a framework for understanding why alterations in chloroplast lipid metabolism become so widely manifested across chloroplast architecture, mesophyll differentiation, leaf morphology, and environmentally responsive growth.

### Light reveals altered developmental plasticity

The strong dependence of the KASIIox phenotype on illumination further supports this interpretation. KASIIox seedlings were comparatively similar to controls during dark growth, whereas developmental abnormalities became pronounced under illumination. Moreover, under low light, control and SSI2ox plants displayed the characteristic adjustment of petiole growth relative to leaf expansion, whereas KASIIox plants remained compact. Thus, the KASII‐associated metabolic state appears particularly consequential when developing tissues must respond to changing light conditions.

Light imposes substantial structural as well as metabolic demands on developing cells. Chloroplast differentiation, thylakoid biogenesis, photosynthetic activity, cellular expansion, and leaf architecture must all be coordinated as plants transition into and adapt to illuminated environments (Casal, 2012; Franklin, 2008; Franklin & Quail, 2009; Legris et al., 2019). The light dependence of the KASIIox phenotype may therefore reflect an increased requirement for a specific membrane composition when photosynthetic tissues undergo rapid structural and metabolic remodeling. From this perspective, the inability of KASIIox plants to execute a characteristic low‐light growth response may represent a limitation in developmental plasticity rather than simply a defect in light perception.

The behavior of phyB and PIF4 is particularly informative in this regard. KASIIox plants displayed altered abundance of these regulators, including elevated PIF4; yet this was not accompanied by the elongation response normally associated with PIF4 activity under low‐light conditions. This apparent disconnect may be more informative than the change in PIF4 abundance itself. It suggests that accumulation of a growth regulator may be insufficient when the metabolic, structural, or cellular context required to execute its developmental output has been altered. Plastidial fatty‐acid metabolism may therefore intersect with light‐responsive development downstream of, or in parallel with, canonical photoreceptor signaling rather than acting simply through changes in individual signaling components (Casal, 2012; Franklin, 2008; Legris et al., 2019). Analysis of downstream PIF4‐responsive transcriptional programs will be required to distinguish among these possibilities.

### GA responsiveness in the context of an altered developmental state

The response to manipulation of GA availability provides a second example of an apparent separation between cellular responsiveness to stimulus and developmental output. Exogenous GA exacerbated KASIIox leaf abnormalities, whereas inhibition of GA biosynthesis with PAC partially restored aspects of leaf morphology, chloroplast size, palisade organization, and mesophyll continuity. These conceptually consistent responses raise the possibility that GA abundance or reception by cellular regulators would be altered in KASIIox plants; however, molecular analyses argued against simple interpretation.

Bioactive GAs and GA intermediates were below the detection limit under our experimental conditions, preventing conclusions regarding endogenous GA abundance. However, RGA1, a DELLA protein whose stability is regulated by GA (Nakajima et al., 2006; Ueguchi‐Tanaka et al., 2005), responded to GA and PAC as expected in KASIIox and control lines, however, its normalized abundance did not differ significantly between these lines. Thus, the developmental phenotype cannot readily be explained by a constitutive difference in RGA1 abundance or by a straightforward disruption of canonical GA reception and signaling.

Nevertheless, the downstream transcriptional response to GA manipulation was not identical in KASIIox and control plants. Lower basal expression of GA3ox1 and GA20ox2 and the stronger GA‐induced response of GASA6 indicate that the KASIIox‐associated metabolic state produces altered engagement of some GA‐responsive genes. Notably, PAC caused expression of GA‐biosynthetic transcripts in KASIIox and control plants to converge, paralleling the partial morphological recovery observed under inhibition of GA biosynthesis. These findings establish an association between KASII‐dependent metabolic remodeling and altered GA‐associated transcriptional responses, but they do not distinguish whether this relationship reflects changes in GA metabolism, signaling activity, downstream transcriptional regulation, or the broader physiological state of KASIIox tissues.

One interpretation consistent with these observations is that the membrane and cellular state generated by sustained KASII expression alters the developmental context in which growth‐promoting signals operate. GA can promote cellular expansion only if cells possess the structural and metabolic capacity to expand while preserving organelle and tissue organization. In KASIIox leaves, that capacity may already be constrained by altered membrane composition, chloroplast organization, or associated changes in cellular physiology. Increasing growth pressure with exogenous GA could therefore accentuate a mismatch between cellular expansion and tissue organization, whereas restricting GA biosynthesis with PAC could reduce these demands and permit partial restoration of tissue architecture. This model does not require KASII to act directly within the GA pathway and remains to be experimentally tested.

### Metabolic state as a context for developmental signaling

The convergence of the light‐ and GA‐associated phenotypes suggests a broader principle emerging from this study: developmental signals operate within a metabolic and structural context that can influence whether their outputs are successfully executed. KASIIox plants retain components of both light‐ and GA‐responsive pathways, yet the corresponding morphological outputs are altered. Plastidial fatty‐acid metabolism may consequently contribute to what could be considered developmental competence, the physiological capacity of a cell or tissue to translate regulatory information into coordinated growth, differentiation, and environmental adaptation.

This framework differs from a model in which KASII acts directly upstream of a particular hormone or photoreceptor pathway. Instead, KASII‐dependent acyl‐chain remodeling may establish cellular conditions that permit or constrain the execution of multiple developmental programs. Such a model could explain why seemingly distinct outputs including chloroplast architecture, mesophyll differentiation, low‐light acclimation, and responses to GA manipulation are simultaneously affected without requiring KASII to regulate each pathway independently. It also provides a testable framework: restoration of membrane composition or particular lipid species should improve developmental responsiveness if the altered lipid state itself contributes to these phenotypes.

### Why is KASII perturbation different from SSI2?

An important unresolved question is why sustained KASII elevation produced extensive lipid and developmental changes, whereas SSI2 elevation had comparatively limited effects. A key consideration is that SSI2 overexpression did not substantially alter fatty‐acid unsaturation. This could indicate that endogenous SSI2 activity is already sufficient to process the available substrate under the conditions examined, such that increasing SSI2 abundance does not appreciably increase desaturation. Alternatively, increased SSI2 activity may be buffered by compensatory changes in other desaturases or lipid‐homeostatic processes that maintain overall unsaturation within a relatively constrained range. Thus, the contrasting KASIIox and SSI2ox phenotypes should not be interpreted as demonstrating that acyl‐chain elongation is intrinsically more important for development than desaturation. Rather, the two perturbations differed in the extent to which they altered the underlying lipid state: KASII elevation produced substantial remodeling of lipid composition which could not be compensated for, whereas SSI2 elevation did not comparably shift unsaturation.

This distinction may also reflect the metabolic position of KASII. By controlling the conversion of C16 to C18 acyl chains, KASII alters substrates that can subsequently enter multiple lipid biosynthetic pathways. Changes in the availability of these products could therefore propagate across plastidial and extraplastidial lipid pools, consistent with the broad redistribution of lipid classes and molecular species observed in KASIIox plants. In contrast, the relatively modest lipid changes in SSI2ox plants may reflect either limited metabolic control at the SSI2 step or effective homeostatic buffering of increased SSI2 abundance. Determining which explanation applies will require direct measurements of metabolic flux and enzyme activity, together with analysis of other desaturases and organelle‐resolved lipid composition. Such approaches should also help distinguish the primary consequences of KASII‐dependent acyl‐chain remodeling from compensatory responses that emerge during development.

The initial identification of MEcPP‐responsive regions in both KASII and SSI2 adds another dimension to this comparison. Although these observations provided the impetus for examining the developmental consequences of perturbing the two enzymes, their physiological significance remains unresolved. Our current data neither establish direct binding of MEcPP to KASII or SSI2 nor demonstrate that MEcPP regulates their activities in vivo. It will therefore be important to determine biochemically whether MEcPP interacts with either enzyme or whether such interactions alter fatty‐acid metabolism under stress. Establishing this connection could reveal a mechanism through which a stress‐associated plastidial metabolite intersects with lipid homeostasis; however, the developmental conclusions of the present study do not depend on such a mechanism.

## CONCLUSIONS

Collectively, our findings support a framework in which plastidial fatty‐acid metabolism contributes to plant development not simply by supplying membrane building blocks but by helping establish the cellular environment within which organelles, cells, and tissues organize and respond to developmental and environmental cues. The contrasting consequences of KASII and SSI2 elevation indicate that perturbations of plastidial fatty‐acid metabolism are not likely to be developmentally equivalent and suggest that acyl‐chain elongation is particularly consequential for membrane and developmental homeostasis.

Rather than supporting a linear model in which KASII directly controls light or GA signaling, our findings point to a broader relationship between metabolic state and developmental competence. Sustained KASII‐dependent acyl‐chain remodeling is associated with altered membrane composition, chloroplast architecture, tissue differentiation, and the capacity to execute appropriate growth responses to environmental and hormonal cues. Identifying the lipid species, membrane properties, and interorganelle exchanges responsible for these associations will be necessary to establish causality. More broadly, these findings provide a foundation for understanding how specific states of organelle metabolism become integrated with the developmental plasticity that enables plants to coordinate growth with a changing environment.

## MATERIALS AND METHODS

### Transgenic lines and plant growth conditions

*Arabidopsis thaliana* genotypes were all developed in Col‐0 ecotype backgrounds. In this study we utilized the *HDSi* genetic background which contains an inverted‐repeat RNA hairpin ~300 bp in length driven under the pOp6/LhGR dexamethasone (DEX) inducible promoter system (Wielopolska et al., 2005) that targets the 5' exon of the HDS gene (4‐hydroxy‐3‐methylbut‐2‐enyl diphosphate synthase) responsible for the conversion of MEcPP to (E)‐4‐Hydroxy‐3‐methylbut‐2‐enyl pyrophosphate (HMBPP) within the MEP pathway. Upon DEX treatment RNAi silencing of HDS expression leads to the accumulation of HDS substrate MEcPP. The *HDSi* (Jiang et al., 2019), and *HDSi ER‐YFP‐HDEL* (Angelos et al., 2026) lines were published previously. The *HDSi inKASII, HDSi inSSI2, HDSi KASIIox*, and *HDSi SSI2ox* lines were generated by *Agrobacterium* transformation and independent transformants homozygous for single inserts were used in this study. *HDSi KASIIox 1 ER‐YFP‐HDEL*, and *HDSi SSI2ox 1 ER‐YFP‐HDEL* were generated by crossing *HDSi ER‐YFP‐HDEL* with *HDSi KASIIox 1* and *HDSi SSI2ox 1* respectively.

For all experiments using sterile culture, seeds were surface sterilized with a 50% bleach solution and rinsed five times with sterile water before being sown on half strength Murashige and Skoog basal medium (2.16 g L^−1^; PlantMedia #30630058‐4) supplemented with MES (1 g L^−1^, 2‐(N‐morpholino) ethanesulfonic acid), adjusted to pH 5.7, with Phytoagar used as a gelling medium (8 g L^−1^; PlantMedia #40100072‐1). Seeds were cold stratified for 2–3 days at 4°C, and plants grown in a Percival growth chamber (long day, 22°C, photosynthetically active radiation [PAR]: 110 μmol m^−2^ s^−1^ or at 5 μmol m^−2^ s^−1^ as indicated in figure legends). PAR was measured using a VBR‐100 (400–700 nm) and VBR‐300 for UVA (365–400 nm) and far‐red light (730 nM; VABIRA, Zhongshan City, Guangdong, China) to ensure parity in wavelength distribution between 110 and 5 μmol m^−2^ s^−1^.

### Plasmid construction and plant transformation

Estradiol inducible lines expressed constructs containing the XVE cassette driven by the UBQ10 promoter and the β‐EST inducible KASII and SSI2 response cassettes generated from the pMDC series of inducible plasmids (Brand et al., 2006). The UBQ10 promoter was amplified from pCAMBIA1300‐UBQpro‐MCS (Guo et al., 2022) vector using primers 1 and 2 (See Table S3 for all primers) and subcloned into pDONR207 via BP Clonase II reaction (Thermo Scientific #11789020). The UBQ10 driven XVE cassette including terminator was then amplified using primers 3 and 4 and inserted into pMDC221 at the XbaI and PmeI restriction enzyme sites (RE; NEB #R0145S, #R0560S) via NEBuilder Hifi DNA Assembly Master Mix (NEB #E2621S) to generate the pMDC371 vector.

KASII and SSI2 full coding sequences (including signal peptides) were amplified from Col‐0 cDNA and subcloned into pDONR207 vectors via BP Clonase II reaction using primers 5–8. To create KASII‐HIS tagged and SSI2‐FLAG tagged variants, primers 9–12 were generated according to Q5 Site Directed Mutagenesis Kit instructions (NEB #E0552S) and reactions were performed with pDONR207‐KASII and pDONR207‐SSI2 plasmids. pDONR207‐KASII‐HIS and pDONR207‐SSI2‐FLAG were recombined with pMDC371 using LR Clonase II reaction (Thermo Scientific 11791020) to generate β‐EST inducible inKASII‐HIS and inSSI2‐FLAG binary vectors.

To generate constitutive overexpression lines driven by UBQ10 promoter, KASII and SSI2 coding sequences were amplified using primers 13–16 and cloned into the pUCG200 plant binary vector via NEB Hifi Assembly reaction (NEB #E2621S) to generate KASIIox and SSI2ox constructs. All vectors were transformed into *Agrobacterium tumefaciens* strain GV3101 and transformed into the Arabidopsis *HDSi* background using the floral dip method (Clough & Bent, 1998) then screened on hygromycin‐containing ½ MS plates to isolate two independent single‐insert transformants.

### Dexamethasone and β‐estradiol treatments

For standard activation of β‐EST inducible lines, 10 mM β‐EST stock (Sigma‐Aldrich #E8875) in DMSO was diluted to 30 μM in sterile double‐distilled H_2_O (ddH_2_O) and filter sterilized. Nine days after germination, sterile cultured seedlings were sprayed *in situ* using a small handheld pump‐action sprayer that was sterilized with 70% ethanol and dried before use. For each spray treatment, ~1 mL of indicated solution was delivered per sterile culture plate in a laminar flow hood. For sustained β‐EST activation, seeds were plated onto ½ MS plates containing 30 μM β‐EST, then sprayed with 30 μM β‐EST every 3 days thereafter to maintain activation of inducible constructs.

For DEX induction of *HDSi* lines 10 mM stock solutions in DMSO (Sigma‐Aldrich #D4902) were diluted to 15 μM in sterile ddH_2_O and 11‐day‐old sterile cultured seedlings were sprayed using the same method as β‐EST.

Whenever seedlings were sprayed with β‐EST or DEX, control groups that did not receive spray from the relevant inducer at 9‐days (β‐EST) or 11‐days (DEX) were treated with mock spray containing equivalent quantities of DMSO vehicle diluted in sterile ddH_2_O. After treatments, seedlings were returned to the growth chamber until analysis at 14 days.

### Gibberellic acid and Paclobutrazol treatments

Stock solutions of 200 mM gibberellic acid (GA_3_; Sigma‐Aldrich #G7645) dissolved in DMSO, and 100 mM paclobutrazol (PAC; Sigma‐Aldrich APO456788397) dissolved in DMSO, were diluted to working concentrations in ddH_2_O and filter sterilized, yielding 100 μM GA_3_ (0.05% DMSO vehicle), 50 μM PAC (0.05% DMSO vehicle), and 10 μM PAC (0.01% DMSO vehicle) solutions. DMSO control working solution contained 0.05% DMSO, a concentration three times lower than DMSO vehicle control concentrations in a previous study of Arabidopsis membrane rigidity which utilized 1% DMSO as a stressing agent (Orvar et al., 2000). Four days after stratification, sterile cultured seedlings were sprayed *in‐situ* using a small handheld pump‐action sprayer, delivering ~1 mL of each indicated solution per plate in a laminar flow hood. For experiments where seedlings were grown to 21 or 35 days after stratification, spray treatments were repeated 14 days after stratification.

### Quantitative reverse transcriptase PCR and RNA‐seq

For targeted gene expression analysis, total RNA was extracted from 14‐day or 21‐day (Figure 7) old shoot tissues according to the manufacturer's instruction for plant material using Aurum Total RNA Mini Kit (Bio‐Rad #7326820), and reverse transcription of 1 μg of the RNA product was performed using iScript Reverse Transcription Supermix (Bio‐Rad #1708840). Subsequent real‐time PCR (RT‐PCR) was performed using SsoAdvanced Universal SYBR Green Supermix (Bio‐Rad #1725274), and the Arabidopsis reference gene AT4G26410 was used as an internal control (Czechowski et al., 2005). Each of the four biological replicates was derived from an independent pool of seedlings from separate sterile culture plates, and each biological replicate was assayed in 3 technical replicates per RT‐PCR experiment. The primers used for RT‐PCR can be found in Table S3.

### Immunoblot analysis

For relative quantification of KASII‐HIS, SSI2‐FLAG, HDS, and actin protein levels in Figure S2, protein was extracted from independent pools of seedlings for each biological replicate. Sixty mg of tissue was ground in liquid nitrogen in a mortar and pestle and resuspended in 180 μL of ice‐cold phosphate‐buffered saline (PBS) protein extraction buffer, pH 7.4 (100 mM NaCl, 1 mM EDTA, 10% glycerol) with 1× cOmplete Mini, EDTA‐free protease inhibitor (Sigma‐Aldrich #NC0962311) and incubated on ice for 10 min. Samples were centrifuged at 21000 ***g*** for 10 min at 4°C, supernatant transferred to a new tube, then centrifuged again. The cleared supernatant was transferred to a new tube and protein concentrations determined using Bio‐Rad Protein Assay (Bio‐Rad #5000006).

Twenty μg of total protein was resolved on an 8% SDS‐PAGE gel and transferred using an overnight wet‐transfer protocol onto a PDVF membrane (Thermo Scientific #PI88518) at 4°C in a transfer buffer containing 20% methanol. Membranes were blocked in Tris‐buffered saline pH 7.5 with 0.1% Tween‐20 (TBST) containing 3% BSA for 60 min, before being probed with anti‐HDS antibody (Wang et al., 2020), diluted 1:1000 in TBST+ 3% BSA for 1 h. Secondary anti‐rabbit antibodies conjugated to horseradish peroxidase (Sigma‐Aldrich #A0545) were then used at a 1:3000 dilution in TBST+3% BSA and detected by SuperSignal West Extended Duration substrate (Thermo Scientific #PIA34075). Membranes were then stripped of antibodies with a mild stripping solution containing 15 g glycine, 1 g SDS, 10 mL Tween‐20 per liter adjusted to pH 2.2 with HCL. Membranes were washed in stripping solution 2× times at room temperature for 10 min, then washed 2× times for 10 min in PBS pH 7.4, then washed 2× times for 5 min in TBST. Stripping was confirmed by the addition of SuperSignal West substrate. Blots were then probed with α‐HIS primary antibodies (Invitrogen #MA1‐21316) against inKASII‐HIS samples or α‐FLAG primary antibodies (Invitrogen #MA1‐91878) against inSSI2‐FLAG samples at 1:3000 dilutions and detected with anti‐mouse HRP conjugated secondary antibodies (Bio‐Rad #1706516) at a 1:5000 dilution. After a second stripping using the described protocol, the loading control actin was probed using α‐actin primary antibodies (1:5000 dilution; Sigma‐Aldrich #A0480) and anti‐mouse HRP secondary antibodies at 1:5000.

For immunoblots of phyB and PIF4, an alternative extraction buffer with additional phosphatase inhibitors was used as described previously (Fan et al., 2025): 100 mM Tris–HCl pH 7.5, 100 mM NaCl, 5% SDS, 5 mM EDTA, 20% glycerol, 20 mM dithiothreitol, 142 mM β‐mercaptoethanol, 1× cOmplete™ EDTA‐free Protease Inhibitor Cocktail (Sigma‐Aldrich #NC0962311), 2 mM phenylmethylsulfonyl fluoride, 80 μM MG132, 80 μM MG115, 1% phosphatase inhibitor cocktail 3 (Sigma‐Aldrich #P0044), 10 mM N‐ethylmaleimide, 2 mM sodium orthovanadate, 25 mM β‐glycerophosphate disodium salt hydrate, 10 mM NaF, and 0.01% bromophenol blue. Protein extracts were boiled for 10 min, centrifuged at 16000 ***g***, and resolved on a 10% SDS‐PAGE gel before being transferred to a PVDF membrane. Primary antibodies were used at a 1:2000 dilution: anti‐PIF4 polyclonal antibody (Abiocode #R2534‐4), α‐phyB monoclonal antibody (a gift from Drs. Akira Nagatani and Yoshito Oka to the Chen Lab) or α‐actin antibodies (Sigma‐Aldrich #A0480). Secondary antibodies were used at a 1:5000 dilution: HRP‐conjugated goat α‐mouse (Bio‐Rad #1706516) or goat α‐rabbit (Bio‐Rad #1706515).

For RGA1 immunoblots 70 mg of tissue from independent pools of seedlings for each biological replicate were frozen in liquid nitrogen, pulverized and extracted in 140 μL of ice‐cold extraction buffer containing 100 mM sodium phosphate buffer pH 7.4, 100 mM NaCl, 10% glycerol (v/v), 60 mM DTT, 25 mM β‐glycerophosphate, 10 mM N‐ethylmaleimide, 5 mM EDTA, 142 mM β‐mercaptoethanol, 80 μM MG132, 20 μM MG115, 1x cOmplete Mini, EDTA‐free protease inhibitor (Fisher Scientific #PIA32955), 1× PhosSTOP inhibitor (Fisher Scientific #NC0962508), 2 mM sodium orthovanadate (from a 200 mM activated stock solution) and 2 mM PMSF. Samples were extracted for 5 min before being centrifuged at 21000 ***g*** for 5 min at 4°C. Supernatant was removed to a new chilled microcentrifuge tube and centrifugation repeated two times. Protein concentration of the final supernatant was determined using Bio‐Rad Protein Assay, and 25 μg of protein from each sample resolved on an 8% SDS‐page gel and then transferred to a PVDF membrane using an overnight wet‐transfer method with a transfer buffer containing 10% methanol. Membranes were blocked in TBST containing 5% skim milk protein for 1 h at room temperature. The α‐RGA1 primary antibody (AT2G01570; rabbit; Fisher Scientific #NC2877336) and α‐actin primary antibodies (mouse; Sigma‐Aldrich #A0480) were co‐incubated with the membrane in TBST +5% skim milk at 1:2000 and 1:10000 dilutions, respectively, for 1 h, then washed 6 times with TBST for 10 min each, then co‐incubated with goat α‐rabbit‐HRP‐conjugated antibodies (1:5000; Bio‐Rad #1706515) and goat α‐mouse‐FITC‐labeled (1:10000, SeraCare #5230–0307) for 1 h. After 6 washes with TBST (10 min each), membranes were detected by SuperSignal West Extended Duration substrate (Thermo Scientific #PIA34075) and fluorescence in a ChemiDoc MP System (BioRad, Hercules, CA) using automated optimal exposure setting (3 × 3). Optimal blot exposure times for RGA1‐HRP detection using these settings were between 10 and 15 min for each blot replicate.

### mPS‐PI staining

Modified Psuedo‐Schiff Propidium Iodide (mPS‐PI) staining was performed as previously described with some modifications (Truernit et al., 2008). Whole shoot tissues (5–8 plants) were fixed in 4 mL of 5:4:1 (v:v:v) solution of methanol:ddH_2_O:acetic acid and stored at 4°C overnight or up to 1 month. Before staining, chlorophyll was removed by incubation in 4 mL 80% ethanol at 80°C for 3 min, repeated twice with fresh 80% ethanol. Then, 80% ethanol was removed and replaced with 40% ethanol, then ddH_2_O with 20 min incubations in each solution. ddH_2_O was then replaced with 4 mL 1% periodic acid (Sigma‐Aldrich P7875) and incubated at room temperature for 1.5 h. Periodic acid was then removed, and samples were washed with 4 mL of ddH_2_O. Then 2 mL of Schiff reagent (100 mM sodium metabisulphite, 0.15 N HCL) containing 0.5 mg/mL propidium iodide (Fisher Scientific #P1304MP) was added and incubated at room temperature in the dark for 1.5 h. Samples were then rinsed with 4 mL ddH_2_O, then stored in 4 mL of ClearSee (Kurihara et al., 2015) optical clearing solution (made by sequentially dissolving 10 g xylitol, 25 g urea, 15 g of sodium deoxycholate in 30 mL of water, then adding further water to 100 mL and warming to 55°C to completely dissolve). mPS‐PI‐stained samples were stored in the dark in ClearSee at room temperature for a minimum of 2 days before confocal microscopy analysis. Leaf tissues were mounted in fresh ClearSee for microscopy analysis or were submerged in melted 1.5% agarose and manually sectioned by scalpel once cooled to produce transverse cross sections of leaves shown in Figure 6 and Figure S9.

### Laser scanning confocal microscopy

Confocal microscopy experiments were performed using a Zeiss LSM900 inverted microscope (Zeiss, Oberkochen, Germany) equipped with Plan‐NEOFLAUR 10×/NA0.3, Plan‐APOCROMAT 20×/NA0.8, and Plan‐APOCROMAT 40×/NA1.4 oil objectives. Excitation by laser line and emission spectra collected for each fluorophore were as follows: ER‐YFP (ex‐514 nm; em‐400–560 nm), Chlorophyll (ex‐640 nm; em‐645–700 nm), mPS‐PI (ex‐488 nm; em 400–645 nm).

For confocal microscopy of live tissues (including ER‐YFP and chlorophyll), single leaves were removed from treatment conditions and mounted in sterile ddH_2_O with a coverslip, and two separate fields of view were imaged in a *Z*‐series from adaxial epidermis to palisade mesophyll in 1 μm intervals.

### Transmission electron microscopy

*Arabidopsis* seedlings were grown as previously specified. The third true leaf was collected 13 days after germination and immediately processed for transmission electron microscopy (TEM). Samples were prepared using standard chemical fixation and *en bloc* heavy‐metal staining following established protocols (Wickramanayake & Czymmek, 2023), with modifications. Briefly, leaf tissue was rapidly placed into cassettes and chemically fixed in 2% paraformaldehyde (EMS #15710), 2% glutaraldehyde (EMS, cat. no. 16020), 0.05% malachite green (Sigma‐Aldrich #M9015), 0.1 M sodium cacodylate buffer pH 7.4 (EMS #11652), and 0.01% Tween‐20 (Sigma‐Aldrich #P1379). Samples were vacuum infiltrated three times for 5 min each at room temperature, and incubated overnight at 4°C. After washing three times in 0.1 M sodium cacodylate buffer, tissues were post‐fixed for 4 h at room temperature in 2% osmium tetroxide (Sigma Aldrich #75632) prepared in 0.1 M sodium cacodylate buffer, followed by three rinses in 3D‐Deionized double‐distilled water (CAS 7732‐18‐5). Next, samples were incubated in 1% aqueous uranyl acetate (SPI‐Chem CAS 6159‐44‐0) overnight at 4°C, followed by incubation at 50°C for 2 h and three ddH_2_O rinses. Samples were then stained with lead aspartate prepared from L‐aspartic acid (Sigma‐Aldrich #A8949) and lead nitrate (Sigma‐Aldrich #203580), adjusted to pH 5.5, and incubated at 60°C for 2 h before rinsing three times in water.

Samples were dehydrated through a graded cold acetone series (25–50–75–95–100%, EMS #10015) and incubated in 100% acetone at 4°C. Dehydrated tissues were treated twice with 100% propylene oxide (EMS #20401) and infiltrated with increasing concentrations of Quetol resin (Quetol 651‐NSA kit; EMS #14640) in propylene oxide (3:1, 2:1, 1:1, 1:2,1:3 (v/v) propylene oxide:Quetol). Final infiltration was performed in 100% Quetol supplemented with DMP‐30, then samples were embedded in flat molds and polymerized at 60°C for 48 h.

Sections of 70 nm thickness were obtained using a Leica Ultracut UCT (Leica Microsystems Inc., Wetzlar, Germany) and mounted on Formvar/Carbon‐coated gold slot grids (EMS #FCF2010‐AU‐SB). TEM was carried out on a Talos 120C G2 microscope (Thermo Fisher Scientific, Waltham, MA, USA) housed in the Advanced Bioimaging Laboratory at the Danforth Center. Imaging was performed at an accelerating voltage of 120 kV using a Ceta 16 M 4 k × 4 k CMOS camera.

### LC–MS quantification of MEcPP

The quantitative extraction of MEcPP was performed as previously described (Xiao et al., 2012). Approximately 50 mg of tissue pulverized in liquid nitrogen was resuspended in 500 μL of extraction solution (10% methanol and 1% acetic acid) containing 2 μM deuterated MEcPP for 30 min at 4°C. Plant extracts were centrifuged twice at 21000 ***g***, 4°C, and 100 μL of the clear supernatant was frozen in liquid nitrogen, lyophilized till dry, then subsequently reconstituted in 500 μL acetonitrile:10 mM ammonium acetate (80:20, v/v).

A Dionex Ultimate 3000 binary RSLC system was used with an Accucore‐150‐Amide‐HILIC column (150 × 2.1 mm; particle size 2.6 μm; Thermo Scientific, Waltham, MA, USA) with a guard column containing the same column matrix. Inject volume was 2 μL, and a gradient elution was performed with acetonitrile (A) and 10 mM ammonium acetate pH 8.0 (B) as the mobile phases. The separation was conducted using the following gradient profile (t (min), %A, %B): (−2, 90, 10), (0, 90, 10), (12, 30, 70), (15, 30, 70), (16, 90, 10), (22, 90, 10). Flow rate was maintained at 280 μL/min. The column was kept at 35°C.

A Q‐Exactive Focus mass spectrometer (Thermo Scientific, Waltham, MA, USA) with a heated electro spray ionization source was used to perform LC–MS analysis. Mass spectra were acquired in negative mode with the following parameters: spray voltage, 3.50 kV; sheath gas flow rate 50, auxiliary gas flow rate 14, sweep gas flow rate 2, capillary temperature at 275°C, S‐lens RF level 100 and auxiliary gas heater temperature at 275°C. A quality control sample was generated by pooling equal aliquots of each sample and analyzed periodically to monitor system stability and performance. Samples were analyzed in random order.

The compound of interest was identified by accurate mass measurements (MS1, Thermo Trace Finder Software), retention time, and mass transitions of purified standard and extract matrixes. Calibration curve from 0.024 to 100 μM of MEcPP to its deuterium‐labeled internal standard was constructed by integrating the area under the peak and plotting the ratios as a function of analyte concentration with regression coefficient of (*R* ^2^) ≥0.98. Absolute concentrations of MEcPP (nmol g^−1^ FW) were quantified using deuterated MEcPP as internal standard and normalized to tissue FW.

### Relative quantification of Phytohormones by LC–MS

Extraction of phytohormones from independent pools of seedlings for each biological replicate was performed as previously described with small modifications (Šimura et al., 2018). Briefly, 30 mg of frozen tissue was pulverized in liquid nitrogen and 1 mL of 50% HPLC grade acetonitrile was added. Tissue was extracted on ice for 1 h and mixed by vortex every 20 min during the extraction. Samples were centrifuged at 21000 ***g***, 4°C, for 10 min, and the supernatant transferred to a new chilled microcentrifuge tube. Centrifugation step was repeated an additional time, and supernatant transferred to a new tube. Sample tissue was reextracted with an additional 0.5 mL of 50% acetonitrile and combined with the previous extract. For each sample, solid phase extraction was performed on a Waters Oasis HLB cartridge (30 mg sorbent; Fisher Scientific #50‐785‐991) equilibrated with 2 mL of 100% HPLC grade methanol, then 2 mL of HPLC grade H2O, and then 2 mL of 50% acetonitrile (all solutions kept ice cold). Combined supernatants were added to the cartridge and the clear, uncolored flow through collected. Flow through for each sample was frozen in liquid nitrogen and then lyophilized to dryness. Lyophilized samples were reconstituted in 40 μL 50% acetonitrile.

Separation was performed on a Waters I‐Class UPLC equipped with a T3 C18 column (100 mm × 2.1 mm; 1.8 μM; Waters, Milford, MA, USA). Inject volume was 2 μL, and a gradient elution was performed with 0.1% Formic Acid in H_2_O (A) and 0.1% Formic Acid in acetonitrile (B). The separation was conducted using the following gradient profile (t (min), %A, %B): (0, 99.9, 0.1), (1, 99.9, 0.1), (6, 45, 55), (7, 0, 100), (8, 0, 100), (8.5, 99.9, 0.1), (13, 99.9, 0.1). Flow rate was maintained at 300 μL/min. The column was kept at 45°C.

A Waters TQ‐XS triple quadrupole mass spectrometer was operated in selected reaction monitoring mode (Waters, Milford, MA, USA). Source and de‐solvation temperatures were 150°C and 600°C, respectively. De‐solvation gas was set to 1100 L/h and cone gas to 150 L/h. Collision gas was set to 0.15 mL/min. All gases were nitrogen except the collision gas, which was argon. The capillary voltage was 1 kV. A quality control sample was generated by pooling equal aliquots of each sample and analyzed periodically to monitor system stability and performance. Samples were analyzed in random order.

Detected phytohormones were integrated using Slyline‐Daily software and values normalized to tissue weight extracted (MacCoss Lab, Seattle, WA).

### Limited proteolysis mass spectroscopy analysis

Limited proteolysis mass spectroscopy (LiP‐MS) analysis was performed as previously described (Schopper et al., 2017). *HDSi* seedlings were harvested 72 h (72‐h) after treatment, and control seedlings (0‐h) were harvested just prior to DEX treatment from the same cohort of seedlings. Seedlings were ground in a mortar and pestle in the presence of a freshly prepared native lysis buffer (20 mM HEPES, 150 mM KCl, and 10 mM MgCl_2_), decanted into a centrifuge tube and centrifuged for 10 min at 21000 and 4°C. Supernatant was transferred to a new tube and the centrifugation step was repeated. Protein concentration was measured using the Bio‐Rad Protein Assay (Bio‐Rad #5000006) and diluted to 1 mg/mL.

For each protein extract, a 100 μg aliquot was brought to a temperature of 25°C in a water bath and proteinase K (Sigma‐Aldrich #P2308) was added to an individual sample in a 1:100 ratio (wt/wt) and digested for exactly 1 min then inactivated by boiling in a water bath for 5 min. Each sample was digested and boiled prior to digesting the next sample for precise timing. Boiled samples were cooled to room temperature and sodium deoxycholate added from a 10% (wt/vol) stock to a final concentration of 5% (wt/vol). Digested and undigested aliquots were then subjected to cysteine reduction by addition of DTT to a final concentration of 12 mM (incubated 30 min at 37°C); alkylation by addition of iodoacetamide (Sigma‐Aldrich #I1149) to a concentration of 40 mM (incubated 45 min at 25°C); lysyl endopeptidase digestion (Wako Pure Chemical Industries #129‐02541; 1:100 ratio peptidase/substrate; 4 h at 37°C); dilution to 1% deoxycholate by addition of 4 volumes of 100 mM ammonium bicarbonate; and an overnight trypsin digestion (Promega #V5113; 1:100 ratio trypsin/substrate; overnight at 37°C). The next day digestion was stopped by addition of formic acid to a final concentration of 2% to precipitate deoxycholate by centrifugation. Peptides were desalted using Sep‐Pak Vac cartridges (Waters #WAT054960) according to manufacturer's instruction, solvent evaporated and peptides resuspended in 0.1% (vol/vol) formic acid. Shotgun proteomics, including peptide separation, LC–MS/MS detection, and processing of raw data using MaxQuant version (2.1.4.0) were performed as described previously (Angelos et al., 2026). To identify candidate peptides with differential abundances in proteinase K + trypsin digested samples with high MEcPP (72 h DEX treatment) compared to controls (0 h), log_2_ fold change in average label‐free quantification intensities of individual peptides was determined and significance tested for each peptide (*P*‐value ≤0.05). Total protein abundance in trypsin‐only digested samples was subjected to the same significance testing and candidate peptides of altered abundance in proteinase K digested samples were considered valid only if total protein abundance of the whole protein was not significantly different (*P*‐value ≥0.05).

### Site specific molecular docking simulation

The AlphaFold protein structural database was used to obtain the predicted structures of KASII (UnitProt—Q9C9P4, source organism—Arabidopsis thaliana) and SSI2 (UnitProt—O22832, source organism—Arabidopsis thaliana) as the receptor models. These predicted structures were selected based on high pLDDT (predicted local distance difference test) scores. These protein structures were refined by using molecular dynamics (MD) simulation. To perform the refinement through simulation, the transit peptides were first removed. Then each protein structure was placed in an orthorhombic box (dimension: 87 Å × 75 Å × 60 Å) where a periodic boundary condition was applied. The protein structures were solvated at physiological condition (pH = 7.4, explicit water solvent density = 0.997 g/mL) and counter ions (Na + and Cl‐) were assigned to neutralize the total system. The MD simulation was conducted at 298 K with a 2 fs (femtosecond) timestep. While conducting the simulation, the YASARA2 force field was applied within an 8.0 Å non‐bonding cut‐off distance covering short‐ and long‐range interactions. The trajectories were saved at every 25 ps (picosecond), yielding 20 snapshots per structure. Further, simulated annealing run was applied for post‐run selection, and the best conformer was selected based on lowest energy criteria. The chosen conformers of KASII and SSI2 were used to perform molecular docking with selected ligands. For ligand preparation, MEcPP (2‐C‐Methyl‐d‐erythritol‐2,4‐cyclopyrophosphate) was prepared by geometry optimization to perform molecular docking with the refined protein structures.

The PyRx Virtual Screening Tool (Dallakyan & Olson, 2015) was used to perform site‐specific docking between structural proteins and ligand. The grid boxes were focused and defined specifically on peptide residues based on LIP assay where the ligands can bind. For KASII, the grid center was defined at *X* = 5.432, *Y* = 0.140, *Z* = −12.085 with dimensions of *X* = 20.0 Å, *Y* = 20.0 Å, *Z* = 12.0 Å. In case of SSI2, the grid center was set to *X* = 7.052, *Y* = −4.262, *Z* = −2.077 with grid box dimensions of *X* = 28.0 Å, *Y* = 21.0 Å, *Z* = 30.0 Å. These grid box parameters ensured the site specificity for enabling comprehensive ligand sampling. Three times docking were conducted to validate the reproducibility of results. Further, the molecular interactions of metabolite‐enzyme (ligand‐protein) complex structures were analyzed in ChimeraX visualization program (Goddard et al., 2018).

### LC–MS/MS quantification of lipids

Lipids were extracted from *HDSi* control plants and overexpressing lines using a boiling water protocol described previously (Gasulla et al., 2013). Glassware including glass Pasteur pipettes used throughout the procedure were new or washed with a 2:1 chloroform: methanol solution and dried prior to use. Plant shoots (5–10 individual plants per biological replicate) were harvested and immediately placed in 5 mL of boiling LCMS grade H_2_O (Fisher Scientific #W64) in a glass culture tube for 20 min to inactivate endogenous lipases, then allowed to cool to room temperature.

To extract lipids from boiled tissues, as much water as possible was removed from the glass tube and 1 mL of a 1:2 chloroform: methanol solution was added, then the sample was vortexed. After a 2 min room temperature incubation, the extraction solution was transferred to a secondary glass tube. This extraction was sequentially repeated with 1 mL of 2:1 chloroform: methanol, and then 1 mL of chloroform. After the extraction solutions were removed, remaining extracted tissues were dried and weighed.

To the 3 mL of extraction solution, 0.75 mL of 300 mM ammonium acetate dissolved in LCMS grade H_2_O was added and the samples vortexed 2× for 10 s each. Then chloroform and aqueous layers were allowed to separate and chloroform layer carefully extracted to a glass screw‐capped vial preloaded with a Teflon septum (Thermo Scientific #03‐452‐498). Lipid extracts were then dried by continuous N_2_ gas stream and glass vials containing the dried lipids purged of ambient air with N_2_ gas for storage until processed for quantification.

Dried lipid samples were resuspended in volumes in Q‐TOF solvent (methanol, chloroform, ammonium acetate 300 mM; 665:300:35 ratio) determined by dried weight of extracted tissues. Aliquots of resuspended lipids (20 μL) were diluted to 200 μL in Q‐TOF solvent with an internal standard mixture containing di14:0‐PC, di20:0‐PC, di14:0‐PE, di20:0‐PE, di14:0‐PG, di20:0‐PG, di14:0‐PA and di20:0‐PA; 0.03 nmol di14:0‐PS and di20:0‐PS; 0.3 nmol of 34:0‐PI; 0.15 nmol 34:0‐MGDG, 0.10 nmol 36:0‐MGDG; 0.2 nmol 34:0‐DGDG, 0.39 nmol 36:0 DGDG; 0.4 nmol SQDG obtained from Avanti Polar Lipids (Alabaster, AL, USA) or Larodan (Solna, Sweden).

Quantification was performed on a Q‐TOF 6540 (Agilent Technologies; Santa Clara, CA, USA) and samples injected at a flow rate of 1 μL/min using a nanospray ion source HPLC Chip/MS 1200 with infusion chip (Agilent Technologies; Santa Clara, CA, USA). Lipids were ionized using a fragmentor voltage of 270 V. Fragmentation energies used were described previously (Gasulla et al., 2013), and data processed using the Mass Hunter Workstation software (version B.02.00). After extraction of neutral loss or precursor ion scanning data from the MS/MS fragment spectra nmol values were calculated using linear regression of the internal standards. Molar (nmol) values were converted to mol % for each biological replicate. Statistically significant differences between genotype and treatment groups compared to HDSi controls were determined by Welch's *t*‐tests with FDR correction applied for multiple comparisons (Table S2).

### Statistical analysis

Quantification of morphological traits from photographs and confocal images was performed in ImageJ (Fiji). As a measure of leaf size, leaf width rather than projected leaf area was used because leaf angles varied among genotypes and treatments, particularly with increasing paclobutrazol concentrations. Projected area was strongly affected by leaf lamina orientation relative to the camera, whereas leaf width provided a more accurate representation of leaf size estimates from *in situ* images.

For non‐omics experiments (qPCR gene expression, leaf width, shoot fresh weight, root length, metabolite quantification), statistical analysis was performed using previously published methods (Angelos & Brandizzi, 2022). One‐way, two‐way, or three‐way type II or type III ANOVA were conducted where relevant, and assumptions of ANOVA model's normality and homogeneity of variance tested by Shapiro–Wilk, Breusch‐Pagan, and Levene's tests respectively. If assumptions were violated (*P* < 0.05), log transformations or Box‐Cox transformations were applied and ANOVA rerun. If transformed datasets still violated the assumptions tests then weighted least squares regression was applied to the ANOVA model, which thereafter passed residual analysis. For the appropriate models post hoc pairwise comparisons with Tukey adjustment were used (CRAN R package: *emmeans*). Summarized statistical results from these datasets are found in graphical representations with full description of statistical analysis performed and results in Table S1.

## Author Contributions

KD and EA designed the research and analyzed the data. KD prepared the manuscript. EA produced the figures and edited the manuscript. EA performed most of the experiments with assistance from AB, and from MG who performed metabolite quantification. KG and PD performed the LC–MS/MS quantification of lipid species. DF and MC performed and provided analysis for phyB and PIF4 blots. AZ and TBS performed and provided analysis for TEM imaging. HC generated the SSI2ox construct and SSI2ox‐2 plant line. LZ, JC, and BZ designed, performed, and analyzed the LiP‐MS experiments.

## Conflict of Interest

The authors declare no competing interests.

## Dedication

This work is dedicated to the memory of our dear colleague and co‐author, Professor Meng Chen, whose untimely passing occurred during the preparation of this manuscript. We deeply mourn his loss and honor his scientific contributions, collegiality, and lasting impact on our community.

## Supporting information


**Figure S1.** MEcPP associates with discrete peptide regions in plastidial fatty‐acid enzymes KASII and SSI2. (A) Volcano plot of peptides identified by LiP‐MS following MEcPP treatment, showing changes in peptide abundance relative to untreated controls. Blue and red dots represent peptides with decreased and increased abundance, respectively. MEcPP‐responsive peptides derived from SSI2 and KASII are highlighted in magenta and cyan, respectively. Differentially abundant peptides were identified based on the indicated fold change (|log_2_ FC| ≥1) and statistical significance thresholds. (B) Summary of enriched peptides identified in SSI2 and KASII, including peptide sequence, amino acid position, fold enrichment (log_2_ FC), and *P*‐values. Two peptide regions in SSI2 (aa 99–119 and aa 301–312) and one region in KASII (aa 356–376) displayed significant enrichment in MEcPP affinity assays. (C) Structural mapping of the enriched KASII peptide region (aa 356–376; yellow) onto the predicted three‐dimensional KASII model. The peptide localizes within the KAS catalytic subdomain in close proximity to the docked MEcPP ligand (magenta). Right panel: enlarged view highlighting the predicted interaction near Phe358, with ligand–peptide distances indicated. (D) Structural mapping of enriched SSI2 peptide regions onto the predicted SSI2 structure. The peptide spanning aa 301–312 (yellow) localizes adjacent to the predicted MEcPP‐binding cavity. Right panel: close‐up view highlighting surrounding residues (Met303, Pro304, and Ala305) that define the predicted interaction environment, with ligand–peptide distances indicated.
**Figure S2.** Inducible expression of KASII or SSI2 does not alter MEcPP accumulation or ER stress signaling, whereas prolonged β‐estradiol treatment impairs seedling growth. (A) Representative images of *Arabidopsis thaliana* control (HDSi), inducible KASII (HDSi inKASII; lines 1 and 2), and inducible SSI2 (HDSi inSSI2; lines 1 and 2) seedlings grown under mock conditions or treated with dexamethasone (DEX; 72 h) to induce HDS silencing, β‐estradiol (β‐EST; 96 h) to induce KASII or SSI2 expression, or combined β‐EST + DEX treatment, as indicated. (B) Quantification of MEcPP levels in the indicated genotypes and treatments. Inducible expression of KASII or SSI2 does not substantially alter MEcPP accumulation under either mock or DEX‐treated conditions. Box plots show median and interquartile range, with points representing individual biological replicates. Different letters indicate statistically significant differences among treatments (*n* = 4; three‐way ANOVA, *P* ≤ 0.05). (C, D) Immunoblot analyses confirming inducible accumulation of KASII–HIS (C) and SSI2–FLAG (D) following β‐EST treatment, together with reduced HDS protein abundance upon DEX treatment. ACTIN was used as a loading control; asterisks indicate non‐specific bands. Lanes represent independent biological replicates. (E) Relative transcript abundance of the ER stress markers *BIP3* and *sbZIP60* measured by RT–qPCR in the indicated genotypes and treatments. β‐EST treatment suppresses DEX‐induced activation of both markers across all backgrounds, indicating that this response is independent of KASII or SSI2 induction. Data are shown relative to mock‐treated HDSi controls. Box plots indicate median and interquartile range, with points as individual biological replicates representing measurements from RNA sourced from independent pools of shoot tissue; different letters denote statistically significant differences (*n* = 4; three‐way ANOVA, *P* ≤ 0.05). (F) Representative seedlings grown under mock or prolonged β‐EST treatment. Sustained β‐EST exposure inhibits seedling growth across all genotypes, with more pronounced growth defects observed in KASII‐ and SSI2‐expressing lines.
**Figure S3.** Constitutive overexpression of KASII and SSI2 elevates transcript abundance with minimal effects on primary root growth. (A) Relative transcript abundance of *KASII* (left) and *SSI2* (right) in control, KASII overexpression (KASIIox 1, KASIIox 2), and SSI2 overexpression (SSI2ox 1, SSI2ox 2) lines. Data are shown as fold change relative to controls. Box plots indicate median and interquartile range, with points as individual biological replicates representing measurements from RNA sourced from independent pools of shoot tissue; asterisks indicate statistically significant differences relative to control (*n =* 4, one‐way ANOVA, *P* ≤ 0.0005). (B) Quantification of 1st true leaf width in the indicated genotypes from images. Box plots indicate mean and interquartile range, with points representing individual biological replicates, asterisks indicate statistically significant differences relative to control (*n* = 80, one‐way ANOVA, *P* ≤ 0.05). (C) Quantification of primary root length in the indicated genotypes. Box plots indicate median and interquartile range, with points representing individual biological replicates, asterisks indicate statistically significant differences relative to control (one‐way ANOVA, *P* ≤ 0.05).
**Figure S4.** Sustained KASII overexpression progressively disrupts vegetative and reproductive plant architecture. (A) Sequential rosette leaf series from control, SSI2 overexpression (SSI2ox 1, SSI2ox 2), and KASII overexpression (KASIIox 1, KASIIox 2) plants harvested at 28, 35, 42, and 49 days after germination (DAG). Dashed boxes indicate representative leaves enlarged in the adjacent panels. Scale bars: 2 cm. (B) Enlarged views of representative rosette leaves from the indicated developmental stages. KASII overexpression produces pronounced defects in leaf symmetry, blade expansion, and chlorophyll accumulation. (C) Representative inflorescence phenotypes at 35 DAG. SSI2ox inflorescences largely resemble controls, although slightly reduced in size, whereas KASIIox plants exhibit compact stature, impaired stem elongation, and altered inflorescence organization. (D) Inflorescence architecture at 49 DAG. KASIIox plants display severe developmental defects, including reduced stem elongation, diminished branching, abnormal floral organization, and impaired silique development. By contrast, SSI2ox plants maintain overall inflorescence architecture similar to controls although formation of mature siliques is highly diminished.
**Figure S5.** Quantification of leaf architecture in plants grown under standard and low light. (A) Quantification of 1st true leaf width in the indicated genotypes from plants grown under standard light conditions (110 μmol m^−2^ s^−1^) for 7 days and then transferred to low‐light conditions (5 μmol m^−2^ s^−1^) or kept in standard light for an additional 5 days. Box plots indicate mean and interquartile range, with points representing individual biological replicates. Different letters indicate statistically significant differences among treatments (*n* = 40; two‐way ANOVA, *P* ≤ 0.05). (B) Quantification of petiole length from 1^st^ true leaves in (A). Box plots indicate mean and interquartile range, with points representing individual biological replicates. Different letters indicate statistically significant differences among treatments (*n* = 40; two‐way ANOVA, *P* ≤ 0.05). (C) For each seedling measured (*n* = 40) the ratio of petiole length (B) to leaf width (A) demonstrates low light adaptation via petiole elongation relative to leaf width. Box plots indicate mean and interquartile range, with points representing individual biological replicates. Different letters indicate statistically significant differences among treatments (*n* = 40; two‐way ANOVA, *P* ≤ 0.05).
**Figure S6.** Independent biological replicate confirms phyB and PIF4 protein responses under standard and low light. (A) Immunoblot analysis of phyB and PIF4 protein abundance in control (C); KASIIox 1, KASIIox 2 (K1, K2); and SSI2ox 1, SSI2ox 2 (S1, S2) seedlings grown under standard light conditions (110 μmol m^−2^ s^−1^) for 7 days and then transferred to low‐light conditions (5 μmol m^−2^ s^−1^) or kept in standard light for an additional 5 days. The *pifq* and *phyB‐9* mutants are included as a control for PIF4 and phyB detection respectively. Actin serves as a loading control. This figure represents a second and third independent biological replicate corresponding to the data shown in Figure 4E. (B) Quantification of phyB and PIF4 (C) band intensity in the indicated genotypes from plants grown under standard light conditions (110 μmol m^−2^ s^−1^) for 7 days and then transferred to low‐light conditions (5 μmol m^−2^ s^−1^) or kept in standard light for an additional 5 days. Box plots indicate mean and interquartile range, with points representing individual biological replicates, asterisks indicate statistically significant differences relative to control (*n* = 3, two‐way ANOVA, *P* ≤ 0.05).
**Figure S7.** KASIIox plants exhibit altered growth responses to gibberellin perturbation. (A) Expanded set of representative 35‐day‐old rosette phenotypes of control and KASIIox plants grown in sterile culture conditions sprayed with sterile GA_3_ (100 μM), paclobutrazol (PAC; 10 or 50 μM), or DMSO control 4 days after germination under long day conditions. Black boxes outline the rosettes shown in Figure 6A. (B) Representative images of 21‐day‐old rosette phenotypes of control and KASIIox plants grown in sterile culture conditions sprayed with sterile GA_3_ (100 μM), paclobutrazol (PAC; 10 or 50 μM), or DMSO control 4 days after germination under long day conditions. Individual leaves from these images are displayed in Figure 6B. (C) Quantification of 1st true leaf width in the indicated genotypes and treatments from plate images. Box plots indicate mean and interquartile range, with points representing individual leaves, different letters indicate statistically significant differences among treatments (*n* = 80; two‐way ANOVA, *P* ≤ 0.05).
**Figure S8.** KASIIox plants exhibit altered palisade cell size in response to gibberellin perturbation. (A) Quantification of the palisade mesophyll cell sizes from 5 randomly selected leaf centers imaged in (Figure 6E). Due to the aberrant morphology of the KASIIox cells in DMSO and GA_3_ treated conditions, palisade mesophyll cells were defined as cells which had observed contact with the adaxial epidermis. Box plot shows median, interquartile range, and points as the quantified value from an individual cell; different letters denote statistically significant differences (two‐way ANOVA, *P* ≤ 0.05). Values of *n* vary from 25 (KASIIox‐ GA_3_ treated) to 72 (KASIIox‐ PAC 50 μM treated), due to the altered abundance of cells in different treatment groups, all *n* values found in Table S1.
**Figure S9.** SSI2ox phenotypic responses to gibberellin perturbation. (A) Representative 21‐day‐old seedlings of control and SSISox plants grown in sterile culture conditions sprayed with sterile GA_3_ (100 μM), paclobutrazol (PAC; 10 or 50 μM), or DMSO control 4 days after germination under long day conditions. (B) Chlorophyll autofluorescence imaging of mesophyll chloroplasts from first true leaves in 21‐day‐old seedlings. Compared with controls, SSI2ox leaves exhibit slightly smaller chloroplasts. Scale bar = 20 μm. A total of 10 leaves per experimental group were sampled from two independent experiments, representative max intensity projections are shown. (C) Quantification of chloroplast size from images shown in (C). From max intensity projections, cross sectional area from twenty mesophyll chloroplasts from 4 randomly selected images were measured (*n* = 80). Box plot shows median, interquartile range, and points as individual chloroplast areas; asterisks denote statistically significant differences from control (two‐way ANOVA, *P* ≤ 0.05). (D) First true leaves of 21‐day‐old plants were fixed, stained with mPS‐PI, and cut to produce adaxial to abaxial transverse cross sections midway between the leaf apex and base. The leaf margin and lamina to left or right of the midvein were then imaged by confocal microscopy (*Z*‐series; 5–10 μm at 1 μm intervals). Scale bar = 60 μm. A total of 10 leaves per experimental group were sampled from two independent experiments, representative max intensity projections are shown. Backgrounds from original images were cropped out to allow for rotation and presentation of the vertical cross sections in the same adaxial‐abaxial orientation on a black background. (E) Quantification of the number of mesophyll cells per image of 5 randomly selected leaf centers imaged in (D). Box plot shows median, interquartile range, and points as the quantified value from an individual leaf; different letters denote statistically significant differences (*n* = 5; two‐way ANOVA, *P* ≤ 0.05). (F) Quantification of the palisade mesophyll cell sizes from 5 randomly selected leaf centers imaged in (D) Box plot shows median, interquartile range, and points as the quantified value from an individual leaf; different letters denote statistically significant differences (*n* = 5; two‐way ANOVA, *P* ≤ 0.05).
**Figure S10.** Relative quantification of phytohormones via LC–MS/MS. (A) Targeted relative quantification of Abscisic acid (ABA), Indole‐3‐acetic acid (IAA), Indole‐3‐carboxylic acid (ICA), jasmonic acid (JA), jasmonoyl‐L‐isoleucine (JA‐ile), salicylic acid (SA), and trans‐zeatin (a cytokinin family hormone) from the same samples. While we were able to detect a wide variety of different phytohormones, we were unable to detect and quantify any bioactive or intermediate metabolite compounds from the gibberellic acid family. Box plots indicate mean and interquartile range, with points representing relative peak area per mg fresh weight for each individual biological replicate (normalized to control sample), different letters indicate statistically significant differences among genotypes (*n* = 4; two‐way ANOVA, *P* ≤ 0.05).
**Figure S11.** Independent biological replicates of RGA1 protein responses. (A) Immunoblot analysis of RGA1 protein abundance in control (C), and KASII overexpression (K1, K2) lines sprayed with the indicated concentrations of GA or PAC. 25 μg of soluble protein were loaded per lane. Actin protein levels served as an internal control. Quantification of RGA1 protein levels from these blots provided in Figure 7C. RGA1 and actin bands of interest are indicated with an asterisk. DMSO lanes indicated with a ‡ in biological replicate 2 contain control protein from biological replicate 1 and 3 to demonstrate similarity in band intensity between blots. (B) Quantification of actin band intensity from all three biological replicates normalized to intensity of control line treated with DMSO vehicle in Figure S11A. Box plot shows median, interquartile range, and points as the quantified and normalized value from an individual band; NS denotes KASIIox experimental groups which are not significantly difference from the control for each treatment. Significant differences denoted by displayed *P*‐values. (*n* = 3; two‐way ANOVA). (C) Ratio of RGA1/actin relative intensities from Figure 7C and Figure S11 respectively. Box plot shows median, interquartile range, and points as the quantified and normalized value from an individual band; NS denotes KASIIox experimental groups which are not significantly difference from the control for each treatment. Significant differences denoted by displayed *P*‐values. (*n* = 3; two‐way ANOVA).

**Table S1.** Summary data and statistical analysis.

**Table S2.** LC–MS/MS quantification of lipid species in *KASIIox* and *SSI2ox*.

**Table S3.** Primers used in this study.

## Data Availability

Requests for further information, reagents, plasmids, or plant lines may be directed to and will be fulfilled by Katayoon Dehesh, corresponding author (kdehesh@ucr.edu). All unique reagents generated in this study are available from the corresponding author upon completion of the Materials Transfer Agreement. Summary data and full results from statistical tests of non‐omics data are provided in Table S1, and lipidomics results in Table S2. No unique code was generated.

## References

[tpj71243-bib-0001] Angelos, E. & Brandizzi, F. (2022) The UPR regulator IRE1 promotes balanced organ development by restricting TOR‐dependent control of cellular differentiation in Arabidopsis. The Plant Journal, 109, 1229–1248. Available from: 10.1111/tpj.15629 PMC8978258 34902186

[tpj71243-bib-0002] Angelos, E.R. , Choi, H.S. , Guo, J. , Zanini, A.A. , Burch‐Smith, T.M. , Snyder, E. et al. (2026) Chloroplast stress signals orchestrate epidermis‐specific remodeling of mitochondria and ER under high light. Advanced Science, 13, e14970. Available from: 10.1002/advs.202514970 PMC13042555 41591266

[tpj71243-bib-0003] Arsovski, A.A. , Haughn, G.W. & Western, T.L. (2010) Seed coat mucilage cells of Arabidopsis thaliana as a model for plant cell wall research. Plant Signaling & Behavior, 5, 796–801. Available from: 10.4161/psb.5.7.11773 PMC3014532 20505351

[tpj71243-bib-0004] Awai, K. , Xu, C. , Lu, B. & Benning, C. (2006) Lipid trafficking between the endoplasmic reticulum and the chloroplast. Biochemical Society Transactions, 34, 395–398. Available from: 10.1042/Bst0340395 16709171

[tpj71243-bib-0005] Benning, C. (2008) A role for lipid trafficking in chloroplast biogenesis. Progress in Lipid Research, 47, 381–389. Available from: 10.1016/j.plipres.2008.04.001 18440317

[tpj71243-bib-0006] Benning, C. , Xu, C.C. , Awai, K. , Lu, B.B. & Gao, J.P. (2007) Lipid trafficking between the endoplasmic reticulum and the chloroplast in the model plant Arabidopsis. FASEB Journal, 21, A37.

[tpj71243-bib-0007] Blanco‐Touriñán, N. , Serrano‐Mislata, A. & Alabadí, D. (2020) Regulation of DELLA proteins by post‐translational modifications. Plant and Cell Physiology, 61, 1891–1901. Available from: 10.1093/pcp/pcaa113 PMC7758031 32886774

[tpj71243-bib-0008] Brand, L. , Hörler, M. , Nüesch, E. , Vassalli, S. , Barrell, P. , Yang, W. et al. (2006) A versatile and reliable two‐component system for tissue‐specific gene induction in Arabidopsis. Plant Physiology, 141, 1194–1204. Available from: 10.1104/pp.106.081299 PMC1533952 16896232

[tpj71243-bib-0009] Cao, D. , Cheng, H. , Wu, W. , Soo, H.M. & Peng, J. (2006) Gibberellin mobilizes distinct DELLA‐dependent transcriptomes to regulate seed germination and floral development in Arabidopsis. Plant Physiology, 142, 509–525. Available from: 10.1104/pp.106.082289 PMC1586041 16920880

[tpj71243-bib-0010] Carlsson, A.S. , LaBrie, S.T. , Kinney, A.J. , von Wettstein‐Knowles, P. & Browse, J. (2002) A KAS2 cDNA complements the phenotypes of the Arabidopsis fab1 mutant that differs in a single residue bordering the substrate binding pocket. The Plant Journal, 29, 761–770. 10.1046/j.1365-313x.2002.01253.x 12148534

[tpj71243-bib-0011] Casal, J.J. (2012) Shade Avoidance. The Arabidopsis Book, 2012. 10.1199/tab.0157 PMC3350169 22582029

[tpj71243-bib-0012] Chan, K.X. , Phua, S.Y. , Crisp, P. , McQuinn, R. & Pogson, B.J. (2016) Learning the languages of the chloroplast: retrograde signaling and beyond. Annual Review of Plant Biology, 67, 25–53. Available from: 10.1146/annurev-arplant-043015-111854 26735063

[tpj71243-bib-0013] Cheminant, S. , Wild, M. , Bouvier, F. , Pelletier, S. , Renou, J.P. , Erhardt, M. et al. (2011) DELLAs regulate chlorophyll and carotenoid biosynthesis to prevent photooxidative damage during seedling deetiolation in Arabidopsis. Plant Cell, 23, 1849–1860. Available from: 10.1105/tpc.111.085233 PMC3123943 21571951

[tpj71243-bib-0014] Clough, S.J. & Bent, A.F. (1998) Floral dip: a simplified method for ‐mediated transformation of. The Plant Journal, 16, 735–743. Available from: 10.1046/j.1365-313x.1998.00343.x 10069079

[tpj71243-bib-0015] Czechowski, T. , Stitt, M. , Altmann, T. , Udvardi, M.K. & Scheible, W. (2005) Genome‐wide identification and testing of superior reference genes for transcript normalization in Arabidopsis. Plant Physiology, 139, 5–17. Available from: 10.1104/pp.105.063743 PMC1203353 16166256

[tpj71243-bib-0016] Dallakyan, S. & Olson, A.J. (2015) In: Hempel, J.E. , Williams, C.H. & Hong, C.C. (Eds.) Chemical Biology: Methods and Protocols. New York: Springer, pp. 243–250.

[tpj71243-bib-0017] Daviere, J.M. & Achard, P. (2013) Gibberellin signaling in plants. Development, 140, 1147–1151. Available from: 10.1242/dev.087650 23444347

[tpj71243-bib-0018] de Lucas, M. , Davière, J.M. , Rodríguez‐Falcón, M. , Pontin, M. , Iglesias‐Pedraz, J.M. , Lorrain, S. et al. (2008) A molecular framework for light and gibberellin control of cell elongation. Nature, 451, 480–484. Available from: 10.1038/nature06520 18216857

[tpj71243-bib-0019] de Souza, A. , Wang, J.Z. & Dehesh, K. (2017) Retrograde signals: integrators of Interorganellar communication and orchestrators of plant development. Annual Review of Plant Biology, 68, 85–108. Available from: 10.1146/annurev-arplant-042916-041007 27813652

[tpj71243-bib-0020] Dormann, P. & Benning, C. (2002) Galactolipids rule in seed plants. Trends in Plant Science, 7, 112–118. Available from: 10.1016/s1360-1385(01)02216-6 11906834

[tpj71243-bib-0021] Fan, D. , Hu, W. , Xu, N. , Seto, E.R. , Lagarias, J.C. , Chen, X. et al. (2025) A multisensor high‐temperature signaling framework for triggering daytime thermomorphogenesis in Arabidopsis. Nature Communications, 16, 5197. Available from: 10.1038/s41467-025-60498-7 PMC12137955 40467614

[tpj71243-bib-0022] Fan, J.L. , Zhai, Z.Y. , Yan, C.S. & Xu, C.C. (2015) Arabidopsis TRIGALACTOSYLDIACYLGLYCEROL5 interacts with TGD1, TGD2, and TGD4 to facilitate lipid transfer from the endoplasmic reticulum to plastids. The Plant Cell, 27, 2941–2955. Available from: 10.1105/tpc.15.00394 PMC4682317 26410300

[tpj71243-bib-0023] Feng, S. , Martinez, C. , Gusmaroli, G. , Wang, Y. , Zhou, J. , Wang, F. et al. (2008) Coordinated regulation of Arabidopsis thaliana development by light and gibberellins. Nature, 451, 475–479. Available from: 10.1038/nature06448 PMC2562044 18216856

[tpj71243-bib-0024] Fernandes, T. , Gonçalves, N.M. , Matiolli, C.C. , Rodrigues, M.A.A. , Barros, P.M. , Oliveira, M.M. et al. (2024) SUMOylation of rice DELLA SLR1 modulates transcriptional responses and improves yield under salt stress. Planta, 260, 136. Available from: 10.1007/s00425-024-04565-1 PMC11549141 39514093

[tpj71243-bib-0025] Franklin, K.A. (2008) Shade avoidance. The New Phytologist, 179, 930–944. Available from: 10.1111/j.1469-8137.2008.02507.x 18537892

[tpj71243-bib-0026] Franklin, K.A. & Quail, P.H. (2009) Phytochrome functions in Arabidopsis development. Journal of Experimental Botany, 61, 11–24. Available from: 10.1093/jxb/erp304 PMC2800801 19815685

[tpj71243-bib-0027] Gallego‐Bartolomé, J. , Minguet, E.G. , Grau‐Enguix, F. , Abbas, M. , Locascio, A. , Thomas, S.G. et al. (2012) Molecular mechanism for the interaction between gibberellin and brassinosteroid signaling pathways in Arabidopsis. Proceedings of the National Academy of Sciences of the United States of America, 109, 13446–13451. Available from: 10.1073/pnas.1119992109 PMC3421204 22847438

[tpj71243-bib-0028] Gasulla, F. , Dorp, K. , Dombrink, I. , Zähringer, U. , Gisch, N. , Dörmann, P. et al. (2013) The role of lipid metabolism in the acquisition of desiccation tolerance in Craterostigma plantagineum: a comparative approach. The Plant Journal, 75, 726–741. Available from: 10.1111/tpj.12241 23672245

[tpj71243-bib-0029] Goddard, T.D. , Huang, C.C. , Meng, E.C. , Pettersen, E.F. , Couch, G.S. , Morris, J.H. et al. (2018) UCSF ChimeraX: meeting modern challenges in visualization and analysis. Protein Science, 27, 14–25. Available from: 10.1002/pro.3235 PMC5734306 28710774

[tpj71243-bib-0030] Grebe, M. , Xu, J. , Möbius, W. , Ueda, T. , Nakano, A. , Geuze, H.J. et al. (2003) Arabidopsis sterol endocytosis involves actin‐mediated trafficking via ARA6‐positive early endosomes. Current Biology, 13, 1378–1387. Available from: 10.1016/s0960-9822(03)00538-4 12932321

[tpj71243-bib-0031] Guo, J. , He, J. , Dehesh, K. , Cui, X. & Yang, Z. (2022) CamelliA‐based simultaneous imaging of Ca2+ dynamics in subcellular compartments. Plant Physiology, 188, 2253–2271. Available from: 10.1093/plphys/kiac020 PMC8968278 35218352

[tpj71243-bib-0032] Hakozaki, H. , Park, J.I. , Endo, M. , Takada, Y. , Kazama, T. , Takeda, Y. et al. (2008) Expression and developmental function of the 3‐ketoacyl‐ACP synthase2 gene in Arabidopsis thaliana. Genes & Genetic Systems, 83, 143–152. Available from: 10.1266/ggs.83.143 18506098

[tpj71243-bib-0033] Hölzl, G. & Dörmann, P. (2019) Chloroplast lipids and their biosynthesis. Annual Review of Plant Biology, 70, 51–81. Available from: 10.1146/annurev-arplant-050718-100202 30786236

[tpj71243-bib-0034] Hornitschek, P. , Kohnen, M.V. , Lorrain, S. , Rougemont, J. , Ljung, K. , López‐Vidriero, I. et al. (2012) Phytochrome interacting factors 4 and 5 control seedling growth in changing light conditions by directly controlling auxin signaling. Plant Journal, 71, 699–711. Available from: 10.1111/j.1365-313X.2012.05033.x 22536829

[tpj71243-bib-0035] Hornitschek, P. , Lorrain, S. , Zoete, V. , Michielin, O. & Fankhauser, C. (2009) Inhibition of the shade avoidance response by formation of non‐DNA binding bHLH heterodimers. EMBO Journal, 28, 3893–3902. Available from: 10.1038/emboj.2009.306 PMC2797054 19851283

[tpj71243-bib-0036] Hu, Y. , Zhou, L. , Huang, M. , He, X. , Yang, Y. , Liu, X. et al. (2018) Gibberellins play an essential role in late embryogenesis of Arabidopsis. Nature Plants, 4, 289–298. Available from: 10.1038/s41477-018-0143-8 29725104

[tpj71243-bib-0037] Huang, X. , Zentella, R. , Park, J. , Reser, L. , Bai, D.L. , Ross, M.M. et al. (2024) Phosphorylation activates master growth regulator DELLA by promoting histone H2A binding at chromatin in Arabidopsis. Nature Communications, 15, 7694. Available from: 10.1038/s41467-024-52033-x PMC11372120 39227587

[tpj71243-bib-0038] Jiang, J. & Dehesh, K. (2021) Plastidial retrograde modulation of light and hormonal signaling: an odyssey. The New Phytologist, 230, 931–937. Available from: 10.1111/nph.17192 33452833

[tpj71243-bib-0039] Jiang, J. , Rodriguez‐Furlan, C. , Wang, J.Z. , de Souza, A. , Ke, H. , Pasternak, T. et al. (2018) Interplay of the two ancient metabolites auxin and MEcPP regulates adaptive growth. Nature Communications, 9, 2262. Available from: 10.1038/s41467-018-04708-5 PMC5995930 29891932

[tpj71243-bib-0040] Jiang, J. , Zeng, L. , Ke, H. , De La Cruz, B. & Dehesh, K. (2019) Orthogonal regulation of phytochrome B abundance by stress‐specific plastidial retrograde signaling metabolite. Nature Communications, 10, 2904. Available from: 10.1038/s41467-019-10867-w PMC6606753 31266952

[tpj71243-bib-0041] Jinpeng, G. , James, G. & Wallis, J.B. (2015) Mutations in the prokaryotic pathway rescue the fatty acid biosynthesis1 mutant in the cold. Plant Physiology, 169, 442–452. Available from: 10.1104/pp.15.00931 PMC4577428 26224803

[tpj71243-bib-0042] Kobayashi, K. (2016) Role of membrane glycerolipids in photosynthesis, thylakoid biogenesis and chloroplast development. Journal of Plant Research, 129, 565–580. Available from: 10.1007/s10265-016-0827-y PMC5897459 27114097

[tpj71243-bib-0043] Kozuka, T. , Kong, S.G. , Doi, M. , Shimazaki, K. & Nagatani, A. (2011) Tissue‐autonomous promotion of palisade cell development by phototropin 2 in Arabidopsis. Plant Cell, 23, 3684–3695. Available from: 10.1105/tpc.111.085852 PMC3229143 21972260

[tpj71243-bib-0044] Kurihara, D. , Mizuta, Y. , Sato, Y. & Higashiyama, T. (2015) ClearSee: a rapid optical clearing reagent for whole‐plant fluorescence imaging. Development, 142, 4168–4179. Available from: 10.1242/dev.127613 PMC4712841 26493404

[tpj71243-bib-0045] Legris, M. , Ince, Y. & Fankhauser, C. (2019) Molecular mechanisms underlying phytochrome‐controlled morphogenesis in plants. Nature Communications, 10, 5219. Available from: 10.1038/s41467-019-13045-0 PMC6864062 31745087

[tpj71243-bib-0046] Leivar, P. & Monte, E. (2014) PIFs: systems integrators in plant development. Plant Cell, 26, 56–78. Available from: 10.1105/tpc.113.120857 PMC3963594 24481072

[tpj71243-bib-0047] Leivar, P. & Quail, P.H. (2011) PIFs: pivotal components in a cellular signaling hub. Trends in Plant Science, 16, 19–28. Available from: 10.1016/j.tplants.2010.08.003 PMC3019249 20833098

[tpj71243-bib-0048] Li‐Beisson, Y. , Shorrosh, B. , Beisson, F. , Andersson, M.X. , Arondel, V. , Bates, P.D. et al. (2013) Acyl‐lipid metabolism. The Arabidopsis Book, 11, e0161. Available from: 10.1199/tab.0161 PMC3563272 23505340

[tpj71243-bib-0049] Munnik, T. & Testerink, C. (2009) Plant phospholipid signaling: “in a nutshell”. Journal of Lipid Research, 50, S260–S265. Available from: 10.1194/jlr.R800098-JLR200 PMC2674723 19098305

[tpj71243-bib-0050] Nakajima, M. , Shimada, A. , Takashi, Y. , Kim, Y.C. , Park, S.H. , Ueguchi‐Tanaka, M. et al. (2006) Identification and characterization of Arabidopsis gibberellin receptors. The Plant Journal, 46, 880–889. Available from: 10.1111/j.1365-313X.2006.02748.x 16709201

[tpj71243-bib-0051] Ohlrogge, J. & Browse, J. (1995) Lipid biosynthesis. Plant Cell, 7, 957–970. Available from: 10.1105/tpc.7.7.957 PMC160893 7640528

[tpj71243-bib-0052] Orvar, B.L. , Sangwan, V. , Omann, F. & Dhindsa, R.S. (2000) Early steps in cold sensing by plant cells: the role of actin cytoskeleton and membrane fluidity. The Plant Journal, 23, 785–794. Available from: 10.1046/j.1365-313x.2000.00845.x 10998189

[tpj71243-bib-0053] Pepelnjak, M. , de Souza, N. & Picotti, P. (2020) Detecting protein‐small molecule interactions using limited proteolysis‐mass spectrometry (LiP‐MS). Trends in Biochemical Sciences, 45, 919–920. Available from: 10.1016/j.tibs.2020.05.006 32475682

[tpj71243-bib-0054] Pidkowich, M.S. , Nguyen, H.T. , Heilmann, I. , Ischebeck, T. & Shanklin, J. (2007) Modulating seed beta‐ketoacyl‐acyl carrier protein synthase II level converts the composition of a temperate seed oil to that of a palm‐like tropical oil. Proceedings of the National Academy of Sciences of the United States of America, 104, 4742–4747. Available from: 10.1073/pnas.0611141104 PMC1838670 17360594

[tpj71243-bib-0055] Sallese, M. , Pulvirenti, T. & Luini, A. (2006) The physiology of membrane transport and endomembrane‐based signalling. The EMBO Journal, 25, 2663–2673. Available from: 10.1038/sj.emboj.7601172 PMC1500860 16763561

[tpj71243-bib-0056] Schopper, S. , Kahraman, A. , Leuenberger, P. , Feng, Y. , Piazza, I. , Müller, O. et al. (2017) Measuring protein structural changes on a proteome‐wide scale using limited proteolysis‐coupled mass spectrometry. Nature Protocols, 12, 2391–2410. Available from: 10.1038/nprot.2017.100 29072706

[tpj71243-bib-0057] Schwechheimer, C. (2011) Gibberellin signaling in plants ‐ the extended version. Frontiers in Plant Science, 2, 107. Available from: 10.3389/fpls.2011.00107 PMC3355746 22645560

[tpj71243-bib-0058] Šimura, J. , Antoniadi, I. , Široká, J. , Tarkowská, D.¡. , Strnad, M. , Ljung, K. et al. (2018) Plant Hormonomics: multiple Phytohormone profiling by targeted metabolomics. Plant Physiology, 177, 476–489. Available from: 10.1104/pp.18.00293 PMC6001343 29703867

[tpj71243-bib-0059] Sun, T.P. & Gubler, F. (2004) Molecular mechanism of gibberellin signaling in plants. Annual Review of Plant Biology, 55, 197–223. Available from: 10.1146/annurev.arplant.55.031903.141753 15377219

[tpj71243-bib-0060] Terashima, I. , Hanba, Y.T. , Tazoe, Y. , Vyas, P. & Yano, S. (2006) Irradiance and phenotype: comparative eco‐development of sun and shade leaves in relation to photosynthetic CO diffusion. Journal of Experimental Botany, 57, 343–354. Available from: 10.1093/jxb/erj014 16356943

[tpj71243-bib-0061] Truernit, E. , Bauby, H. , Dubreucq, B. , Grandjean, O. , Runions, J. , Barthélémy, J. et al. (2008) High‐resolution whole‐mount imaging of three‐dimensional tissue organization and gene expression enables the study of phloem development and structure in Arabidopsis. The Plant Cell, 20, 1494–1503. Available from: 10.1105/tpc.107.056069 PMC2483377 18523061

[tpj71243-bib-0062] Ubeda‐Tomas, S. , Federici, F. , Casimiro, I. , Beemster, G.T.S. , Bhalerao, R. , Swarup, R. et al. (2009) Gibberellin signaling in the endodermis controls Arabidopsis root meristem size. Current Biology, 19, 1194–1199. Available from: 10.1016/j.cub.2009.06.023 19576770

[tpj71243-bib-0063] Ueguchi‐Tanaka, M. , Ashikari, M. , Nakajima, M. , Itoh, H. , Katoh, E. , Kobayashi, M. et al. (2005) GIBBERELLIN INSENSITIVE DWARF1 encodes a soluble receptor for gibberellin. Nature, 437, 693–698. Available from: 10.1038/nature04028 16193045

[tpj71243-bib-0064] Walley, J. , Xiao, Y. , Wang, J.Z. , Baidoo, E.E. , Keasling, J.D. , Shen, Z. et al. (2015) Plastid‐produced interorgannellar stress signal MEcPP potentiates induction of the unfolded protein response in endoplasmic reticulum. Proceedings of the National Academy of Sciences of the United States of America, 112, 6212–6217. Available from: 10.1073/pnas.1504828112 PMC4434691 25922532

[tpj71243-bib-0065] Wang, J.‐Z. , Lei, Y. , Xiao, Y. , He, X. , Liang, J. , Jiang, J. et al. (2020) Uncovering the functional residues of *Arabidopsis* isoprenoid biosynthesis enzyme HDS. Proceedings of the National Academy of Sciences, 117, 355–361. Available from: 10.1073/pnas.1916434117 PMC6955319 31879352

[tpj71243-bib-0066] Wang, J.Z. , Li, B. , Xiao, Y. , Ni, Y. , Ke, H. , Yang, P. et al. (2017) Initiation of ER body formation and indole Glucosinolate metabolism by the Plastidial retrograde signaling metabolite, MEcPP. Molecular Plant, 10, 1400–1416. Available from: 10.1016/j.molp.2017.09.012 PMC6368977 28965830

[tpj71243-bib-0067] Wang, J.‐Z. , Ven, W. , Xiao, Y. , He, X. , Ke, H. , Yang, P. et al. (2022) Reciprocity between a retrograde signal and a putative metalloprotease reconfigures plastidial metabolic and structural states. Science Advances, 8, eabo0724. Available from: 10.1126/sciadv.abo0724 PMC9166295 35658042

[tpj71243-bib-0068] Western, T.L. , Skinner, D.J. & Haughn, G.W. (2000) Differentiation of mucilage secretory cells of the Arabidopsis seed coat. Plant Physiology, 122, 345–356. Available from: 10.1104/pp.122.2.345 PMC58872 10677428

[tpj71243-bib-0069] Wickramanayake, J.S. & Czymmek, K.J. (2023) A conventional fixation volume electron microscopy protocol for plants. Methods in Cell Biology, 177, 83–99. Available from: 10.1016/bs.mcb.2023.04.008 37451777

[tpj71243-bib-0070] Wielopolska, A. , Townley, H. , Moore, I. , Waterhouse, P. & Helliwell, C. (2005) A high‐throughput inducible RNAi vector for plants. Plant Biotechnology Journal, 3(6), 583–590. Available from: 10.1111/j.1467-7652.2005.00149.x 17147629

[tpj71243-bib-0071] Wu, J. , Lightner, J. , Warwick, N. & Browse, J. (1997) Low‐temperature damage and subsequent recovery of fab1 mutant Arabidopsis exposed to 2°C. Plant Physiology, 113, 347–356. Available from: 10.1104/pp.113.2.347 PMC158148 9046588

[tpj71243-bib-0072] Xiao, Y. , Savchenko, T. , Baidoo, E.E.K. , Chehab, W.E. , Hayden, D.M. , Tolstikov, V. et al. (2012) Retrograde signaling by the Plastidial metabolite MEcPP regulates expression of nuclear stress‐response genes. Cell, 149, 1525–1535. Available from: 10.1016/j.cell.2012.04.038 22726439

[tpj71243-bib-0073] Xiao, Y. , Wang, J. & Dehesh, K. (2013) Review of stress specific organelles‐to‐nucleus metabolic signal molecules in plants. Plant science: an international journal of experimental plant biology, 212, 102–107. Available from: 10.1016/j.plantsci.2013.08.003 24094057

[tpj71243-bib-0074] Xu, C.C. , Fan, J.L. , Cornish, A.J. & Benning, C. (2008) Lipid trafficking between the endoplasmic reticulum and the plastid in requires the extraplastidic TGD4 protein. The Plant Cell, 20, 2190–2204. Available from: 10.1105/tpc.108.061176 PMC2553622 18689504

[tpj71243-bib-0075] Xu, Y. , du, J. , Hao, R. , Ma, S. , Ma, Y. , Zhou, G. et al. (2025) Gibberellin signaling regulates pectin biosynthesis in Arabidopsis. Nature Communications, 16, 4065. Available from: 10.1038/s41467-025-59268-2 PMC12044110 40307219

[tpj71243-bib-0076] Yu, C.‐W. , Lin, Y.‐T. & Li, H.‐m. (2020) Increased ratio of galactolipid MGDG: DGDG induces jasmonic acid overproduction and changes chloroplast shape. New Phytologist, 228, 1327–1335. Available from: 10.1111/nph.16766 PMC7689733 32585752

[tpj71243-bib-0077] Zeng, L. , Gomez Mendez, M.F. , Guo, J. , Jiang, J. , Zhang, B. , Chen, H. et al. (2024) Activation of stress‐response genes by retrograde signaling‐mediated destabilization of nuclear importin IMPα‐9 and its interactor TPR2. Molecular Plant, 17, 884–899. Available from: 10.1016/j.molp.2024.04.008 38693693

[tpj71243-bib-0078] Zeng, L. , Wang, J.Z. , He, X. , Ke, H. , Lemos, M. , Gray, W.M. et al. (2022) A plastidial retrograde signal potentiates biosynthesis of systemic stress response activators. The New Phytologist, 233, 1732–1749. Available from: 10.1111/nph.17890 PMC8776617 34859454

[tpj71243-bib-0079] Zentella, R. , Hu, J. , Hsieh, W.P. , Matsumoto, P.A. , Dawdy, A. , Barnhill, B. et al. (2016) O‐GlcNAcylation of master growth repressor DELLA by SECRET AGENT modulates multiple signaling pathways in Arabidopsis. Genes & Development, 30, 164–176. Available from: 10.1101/gad.270587.115 PMC4719307 26773002

[tpj71243-bib-0080] Zentella, R. , Sui, N. , Barnhill, B. , Hsieh, W.P. , Hu, J. , Shabanowitz, J. et al. (2017) The Arabidopsis O‐fucosyltransferase SPINDLY activates nuclear growth repressor DELLA. Nature Chemical Biology, 13, 479–485. Available from: 10.1038/nchembio.2320 PMC5391292 28244988

[tpj71243-bib-0081] Zhao, Y. , Yan, A. , Feijó, J.A. , Furutani, M. , Takenawa, T. , Hwang, I. et al. (2010) Phosphoinositides regulate clathrin‐dependent endocytosis at the tip of pollen tubes in Arabidopsis and tobacco. Plant Cell, 22, 4031–4044. Available from: 10.1105/tpc.110.076760 PMC3027160 21189293

[tpj71243-bib-0082] Zhong, C. , Xu, H. , Ye, S. , Wang, S. , Li, L. , Zhang, S. et al. (2015) Gibberellic acid‐stimulated Arabidopsis6 serves as an integrator of gibberellin, Abscisic acid, and glucose signaling during seed germination in Arabidopsis. Plant Physiology, 169, 2288–2303. Available from: 10.1104/pp.15.00858 PMC4634064 26400990

